# Comprehensive collection of genes and comparative analysis of full-length transcriptome sequences from Japanese larch (*Larix kaempferi*) and Kuril larch (*Larix gmelinii* var. *japonica*)

**DOI:** 10.1186/s12870-022-03862-9

**Published:** 2022-10-04

**Authors:** Kentaro Mishima, Hideki Hirakawa, Taiichi Iki, Yoko Fukuda, Tomonori Hirao, Akira Tamura, Makoto Takahashi

**Affiliations:** 1 Tohoku Regional Breeding Office, Forest Tree Breeding Center, Forestry and Forest Products Research Institute, Forest Research and Management Organization, 95 Osaki, Takizawa, Iwate, 020-0621 Japan; 2 Kazusa DNA Research Institute, 2-6-7 Kazusa-kamatari, Kisarazu, Chiba 292-0818 Japan; 3 Hokkaido Regional Breeding Office, Forest Tree Breeding Center, Forestry and Forest Products Research Institute, Forest Research and Management Organization, 561-1 Bunkyodaimidorimachi, Ebetsu, Hokkaido 069-0836 Japan; 4 Forest Tree Breeding Center, Forestry and Forest Products Research Institute, Forest Research and Management Organization, 3809-1 Ishi, Juo, Hitachi, Ibaraki 319-1301 Japan

**Keywords:** *Larix kaempferi*, *L. gmelinii* var. *japonica*, *Isoform sequencing*, Short-read sequences, Flowering signal-related genes

## Abstract

**Background:**

Japanese larch (*Larix kaempferi*) is an economically important deciduous conifer species that grows in cool-temperate forests and is endemic to Japan. Kuril larch (*L. gmelinii* var. *japonica*) is a variety of Dahurian larch that is naturally distributed in the Kuril Islands and Sakhalin. The hybrid larch (*L. gmelinii* var. *japonica* × *L. kaempferi*) exhibits heterosis, which manifests as rapid juvenile growth and high resistance to vole grazing. Since these superior characteristics have been valued by forestry managers, the hybrid larch is one of the most important plantation species in Hokkaido. To accelerate molecular breeding in these species, we collected and compared full-length cDNA isoforms (Iso-Seq) and RNA-Seq short-read, and merged them to construct candidate gene as reference for both Larix species. To validate the results, candidate protein-coding genes (ORFs) related to some flowering signal-related genes ​were screened from the reference sequences, and the phylogenetic relationship with closely related species was elucidated.

**Results:**

Using the isoform sequencing of PacBio RS ll and the *de novo* assembly of RNA-Seq short-read sequences, we identified 50,690 and 38,684 ORFs in Japanese larch and Kuril larch, respectively. BUSCO completeness values were 90.5% and 92.1% in the Japanese and Kuril larches, respectively. After comparing the collected ORFs from the two larch species, a total of 19,813 clusters, comprising 22,571 Japanese larch ORFs and 22,667 Kuril larch ORFs, were contained in the intersection of the Venn diagram. In addition, we screened several ORFs related to flowering signals (*SUPPRESSER OF OVEREXPRESSION OF CO1: SOC1, LEAFY: LFY, FLOWERING Locus T: FT, CONSTANCE: CO*) from both reference sequences, and very similar found in other species.

**Conclusions:**

The collected ORFs will be useful as reference sequences for molecular breeding of Japanese and Kuril larches, and also for clarifying the evolution of the conifer genome and investigating functional genomics.

**Supplementary Information:**

The online version contains supplementary material available at 10.1186/s12870-022-03862-9.

## Introduction

Coniferous tree species are often dominant in boreal forests where they play an important role in the ecology of forest ecosystems. The genetic and evolutionary characteristics of conifers have been widely studied and numerous comparative studies with other seed-producing plants have been conducted [1]. Conifers are frequently selected for breeding, as they provide important building materials and fuel resources for humans. Further, afforestation also increases carbon storage in forests [2, 3], and is therefore regarded as an effective mitigation measure against climate change [4].

The coniferous genus *Larix* contains ten species and seven varieties with wind-mediated pollen and seed dispersal that are often dominant in the cool-temperate and subarctic forests of the northern hemisphere [5]. Japanese larch (*Larix kaempferi*) is among the most important forestry tree species in northern Japan. More than 500 Japanese larch plus trees were selected from both natural and man-made larch forests, which together cover an area of 1 million ha and account for approximately 10% of the artificial forests in Japan [6]. Japanese larch has suitable characteristic for forestry and was introduced to Hokkaido from the central mountainous region of Honshu since the early part of the last century [7]. However, shortly after Japanese larch was introduced to Hokkaido, the species was adversely affected by vole grazing and dieback disease [7–9]. On the other hand, Kuril larch (*Larix gmelinii* var. *japonica*), which was introduced to Hokkaido from the Kuril Islands and Sakhalin, showed higher resistance to vole grazing than Japanese larch, but the growth was slower than that of Japanese larch. To overcome the disadvantages of the two species, hybrid seedlings of Kuril larch and Japanese larch were produced by artificial interspecific crossing. The hybrid larch, which exhibits heterosis in the form of rapid juvenile growth and high resistance to vole grazing [7–9], is currently one of the most important species in Hokkaido.

Recent advances in sequencing technology and bioinformatics have enabled researchers to perform genome-wide surveys of various economically and/or ecologically important crops (e.g., rice [10], tomato [11], soybean [12], and maize [13]). However, the amount of reference genome information that is available for coniferous species is currently limited to several species (e.g., *Picea abies* [14], *Pinus taeda* [15], *Pinus lambertiana* [16], *Pseudotsuga menziesii* var. *menziesii* [17], and *Pinus tabuliformis* [18], *Larix sibilica* [19], *Sequoia sempervirens* [20], *Larix kaempferi* [21]), mainly because their large genomes and complex genomic structures have hindered the precise elucidation of their genomes. Although a reference genome sequence for the Japanese larch has been published [21] and deposited in GenBank/DDBJ/EMBL (Accession No.: WOXR00000000), genetic information is not available. Compared to crop species, reference genome information for coniferous species has not been used effectively. Although numerous examples of polymorphisms and transcriptome information have been collected for coniferous species based on expressed sequence tag (EST) data, correlations between various traits for molecular breeding and the geographical structure of species have been performed mainly without reference genome sequences (e.g., *Cryptomeria japonica* [22, 23], *Picea glauca* [24, 25], *P. abies* [26, 27], *Pinus thunbergii* [28], *P. taeda* [29–31]).

RNA sequencing (RNA-Seq) has been widely used to construct transcript sequences, such as unigenes, for numerous of plant species, especially, for plant species with large genome size, such as forest trees. Consequently, a large amount of transcriptome data for coniferous species that do not require a reference genome has been collected for various research applications [1, 32–36]. RNA-Seq facilitates the accurate and large-scale sequencing of cDNA and is effective for characterizing genetic models without reference genomes [37]. However, RNA-Seq short-reads are often not full-length transcripts, which means that they require large-scale computational assemblies to reconstruct transcript sequences; there is thus a trade-off between sampling depth and data integrity [38, 39]. Further, constructing full-length transcript sequences using short-reads is often complicated by factors such as mis-assemblies and low coverage of reads. Since short-reads can cause mis-assembly of transcripts, long-read sequencing techniques are considered preferable for reconstructing full-length transcripts. The single-molecule real-time (SMRT) sequencing technology developed by Pacific Biosciences (PacBio Inc.) has facilitated the elucidation of highly accurate long-reads, which can overcome the problems introduced by the short-read approach. In addition, the SMRT sequencing technology has the advantage of producing full-length cDNA sequences and can be used to characterize the structural variation of isoforms derived from alternative splicing [35, 40]. However, due to low coverage, obtaining all of the transcripts using only long-read data would be difficult. Therefore, to efficiently construct exhaustive cDNA sequences, it is desirable to perform a combination of short- and long-read sequencing. To do this, short-read sequences are assembled *de novo* before being merged with long-read sequences using a clustering approaches.

The objective of this study was to establish a comprehensive collection of full-length transcriptome sequences for Japanese larch and Kuril larch which can be applied to molecular breeding of both species and hybrids. To this end, we identified the Japanese larch and Kuril larch reference transcriptome sequences using a combination of long-read (isoform sequence with PacBio RS ll isoform (Iso-Seq)) and short-read (RNA sequence with Illumina (RNA-Seq)) approaches, and the obtained reference transcriptome sequences of both species, which were then compared to clarify their similarities. Moreover, in order to verify the usefulness of the constructed full-length transcriptome sequences, ORFs related to flowering-related signal genes were screened using reference sequences. Then, the sequence similarities of these candidate ORFs and the phylogenetic relationships with closely related species were compared. *Larix* species, they show considerable year-to-year variation in flower production and few consecutive flowering events are typically observed [41]. In addition, mast seedling makes it difficult for forestry managers to formulate efficient tree breeding programs for these species. As a result, we need to elucidate the genetic processes responsible for controlling flowering need to be elucidated using molecular methods, but information on the flowering genes of these species is scarce. The reference sequences constructed in this study shows that these sequences contained the background information that could be used to elucidate the flowering mechanisms of the genus *Larix*.

The collected genetic information will also be used as a transcriptome reference for future research on the genus *Larix*.

## Results

### ORF prediction for the transcriptomes obtained by isoform sequencing (Iso-Seq)

Bulked RNA extracted from three tissue types (cambium, needles, and shoots) was sequenced to achieve wide coverage of the transcriptome using PacBio isoform sequencing for each of the two *Larix* species. A total of 501,286 and 459,268 reads of inserts (ROIs) were generated, with 1,566,511,985 and 1,224,443,307 nucleotides obtained from nine (insert size: 1–2 kb, 2–3 kb, 3–6 kb, 5–10 kb) and ten (1–2 kb, 2–3 kb, 3–4 kb, 4–10 kb) SMRT cells from Japanese and Kuril larches, respectively (Table 1). For Japanese and Kuril larches, the mean read lengths were 3,125 and 2,666 bp, respectively, with 246,070 (49.1%) and 248,940 (54.2%) full-length (FL) ROIs and 242,646 (48.4%) and 246,043 (53.6%) FL non-chimeric ROIs, respectively. After using the Iso-Seq clustering algorithm, (iterative clustering for error correction (ICE)), a total of 91,714 and 83,026 high-quality, polished isoforms and 40,693 and 50,225 low-quality polished isoforms of FL non-chimeric ROIs were obtained by Quiver polishing, respectively. Finally, high-quality, non-redundant isoforms were obtained comprising 79,832 and 66,002 sequences, with lengths ranging from 300–8,880 bp (mean 2,715 bp) and 307–10,117 bp (mean 2,446 bp), respectively. BUSCO analysis revealed that 65.1% and 71.9% of the 1,375 BUSCOs in embryophytes (odb10) were found with completeness in Japanese larch and Kuril larches, respectively (72.9% and 78.6% when fragmented BUSCOs were included).

**Table 1:** Summary of collections of PacificBio transcript isoform data for Japanese larch and Kuril larch

		*Larix kaempferi* (DRA:011937, Experiment: DRX279227)					*Larix gmelinii* var. *japonica* (DRA: 011937, Experiment: DRX279228)				
		1–2 kb (3 cells)	2–3 kb (2 cells)	3–6 kb (2 cells)	5–10 kb (2 cells)	Total	1–2 kb (3 cells)	2–3 kb (3 cells)	3–4 kb (2 cells)	4–10 kb (2 cells)	Total
Read of Insert (ROI)		150,023	122,662	114,064	114,537	501,286	124,921	141,921	102,575	89,851	459,268
Read of bases insert		277,644,841	313,765,905	421,270,147	553,831,092	1,566,511,985	189,871,361	342,758,385	342,431,605	349,381,956	1,224,443,307
Mean read length of insert (bases)		1,850	2,557	3,693	4,835	3,125	1,519	2,415	3,338	3,888	2,666
Mean read quality of insert		0.9236	0.9153	0.8953	0.8579	4	0.9256	0.9241	0.9059	0.8881	4
Mean number of passes		10	8	5	3	26	12	8	5	4	29
Number of filtered short reads of insert		7,192	3,558	1,374	1,075	13,199	6,125	3,233	1,365	1,184	11,907
Number of non-full-length reads of insert		62,498	50,252	51,889	77,378	242,017	49,722	53,965	45,424	49,310	198,421
Number of full-length reads of insert		80,333	68,852	60,801	36,084	246,070	69,074	84,723	55,786	39,357	248,940
Number of full-length non-chimeric reads		78,930	68,352	60,554	34,810	242,646	67,549	84,271	55,564	38,659	246,043
Average full-length non-chimeric read length		1,745	2,608	4,002	5,798	-	1,359	2,445	3,599	4,411	-
Number of consensus isoforms		46,124	43,436	28,889	13,958	132,407	33,508	45,851	29,364	24,534	133,257
Average consensus isoforms read length (bases)		1,753	2,567	4,023	5,246	13,589	1,439	2,583	3,809	4,253	12,084
Number of polished high-quality isoforms		36,281	29,509	17,489	8,435	91,714	26,093	30,438	15,888	10,607	83,026
Number of polished low-quality isoforms		9,843	13,927	11,400	5,523	40,693	7,415	15,410	13,476	13,924	50,225
Non-redundant transcripts		-	-	-	-	79,832	-	-	-	-	66,002
Min. isoform length (bases)		-	-	-	-	300	-	-	-	-	307
Max. isoform length (bases)		-	-	-	-	8,880	-	-	-	-	10,117
Mean isoform length (bases)		-	-	-	-	2,715	-	-	-	-	2,446
BUSCO v3 (odb10; 1,375)	Complete (%)	-	-	-	-	65.1	-	-	-	-	71.9
	Complete and single-copy (%)	-	-	-	-	31.9	-	-	-	-	33.1
	Complete and duplicated (%)	-	-	-	-	33.2	-	-	-	-	38.8
	Fragmented (%)	-	-	-	-	7.8	-	-	-	-	6.7
	Missing (%)	-	-	-	-	27.1	-	-	-	-	21.4

ORF prediction was conducted to obtain high-quality non-redundant isoforms using ANGEL software. For the Japanese and Kuril larches, 80,557 and 67,332 ORFs were predicted, ranging from 145–7,956 bp (mean 1,356 bp) and 146–6,609 bp (mean 1,172 bp), respectively (Table 2). Among the total predicted ORFs, 37,508 (46.6%) and 29,372 (43.6%), were confident ORFs (confident-complete, 5’ partial, 3’ partial, internal), respectively. In the BUSCO analysis, the completeness of the confident ORFs in Japanese and Kuril larches was 46.8% and 52.3%, respectively (Table 3).

**Table 2:** Open reading frame prediction of PacBio transcript isoform data estimated by ANGEL

	*Larix kaempferi*	*Larix gmelinii* var. *japonica*
Number of ORFs	80,557	67,332
Total length (bases)	109,217,543	78,919,963
Average (bases)	1,356	1,172
Maximum (bases)	7,956	6,609
Minimum ((bases)	145	146
N50 (bases)	1,752	1,551
G + C%	44.8	44.5
Category of ANGEL		
Confident	37,508	29,372
Confident-complete	20,886	20,201
Confident-5'partial	16,277	8,866
Confident-3'partial	234	251
Confident-internal	111	54
Likely-NA	9,064	6,910
Suspicious-NA	16,834	15,718
Dumb-complete	16,992	15,187
Dumb-5'partial	11	3
Dumb-3'partial	148	142
BUSCO v3 (odb10; 1,375)		
Complete (%)	63.2	68.2
Complete and single-copy (%)	30.5	32.5
Complete and duplicated (%)	32.7	35.7
Fragmented (%)	7.7	8.1
Missing (%)	29.1	23.7

**Table 3:** Completeness of all confident ORFs (confident-complete, 5’_partial, 3’_partial, internal) and confident-complete ORFs estimated by BUSCO analysis

	*Larix kaempferi* (ANGEL, Confident)	*Larix gmelinii* var. *japonica* (ANGEL, Confident)	*Larix kaempferi* (ANGEL, Confident-complete)	*Larix gmelinii* var. *japonica* (ANGEL, Confident-complete)
Complete (%)	46.8	52.3	35.1	42.4
Complete and single-copy (%)	23.8	26.8	19.9	23.1
Complete and duplicated (%)	23.0	25.5	15.2	19.3
Fragmented (%)	6.5	4.9	4.9	3.9
Missing (%)	46.7	42.8	60.0	53.7

### ORF prediction of the transcriptomes obtained by *de novo* assembly of short-read sequences (RNA-Seq)

The RNA-Seq short-reads sequenced from the two of *Larix* species were assembled to compare the differences between the transcript sequences and their expression. A total of approximately 1 billion raw reads for the two species were generated and the Q20 and Q30 quality score cutoffs and GC contents (%) are shown in Table 4. The *in silico* normalized reads for the Japanese and Kuril larches (182,578,084 reads and 209,039,560 reads, respectively) were applied to the first *de novo* assembly by using Trinity v2.8.5, and 912,369 and 1,133,931 contigs with N50 length of 1,112 bp and 1,016 bp were obtained for the Japanese and Kuril larches, respectively (Tables 4, 5). The trimmed reads were mapped against the contigs, and fragments per kilobase per million fragments mapped (FPKM) values > 1 were selected as “unitranscripts” to remove low-quality transcripts including mis-assemblies. Next, the longest unitranscripts from each gene locus were selected as unigenes. Finally, 58,396 and 36,972 unigene sequences with lengths ranging from 188–17,703 bp (mean 1,171 bp) and 185–18,429 bp (mean 1,334 bp), and N50 lengths of 2,246 bp and 2,474 bp were obtained for Japanese and Kuril larches, respectively (Table 5).

**Table 4:** Summary of collections to short-read of transcript data

		*Larix kaempferi* (LK)							*Larix gmelinii* var. *japonica (*LG)								
Sample name		Total reads	Total read bases	GC (%)	AT (%)	Q20 (%)	Q30 (%)	Accession	Sample name		Total reads	Total read bases	GC (%)	AT (%)	Q20 (%)	Q30 (%)	Accession
LK	GFE33389	144,905,924	14,635,498,324	45.26	54.74	96.43	94.07	DRA: 011937, Experiment: DRX279213	LG	GFF08127	149,916,032	15,141,519,232	45.45	54.55	95.30	92.09	DRA: 011937, Experiment: DRX279223
LK	GFE02991	159,873,888	16,146,262,688	45.22	54.78	95.46	92.34	DRA: 011937, Experiment: DRX279214	LG	GFF03187	178,050,660	17,983,116,660	45.05	54.95	94.61	91.03	DRA: 011937, Experiment: DRX279221
LK	GEF32200	124,904,528	12,615,357,328	45.49	54.51	99.00	97.34	DRA: 011937, Experiment: DRX279215	LG	GFF03199	171,178,302	17,289,008,502	44.79	55.21	95.55	92.45	DRA: 011937, Experiment: DRX279220
LK	GEF32203	127,808,704	12,908,679,104	45.96	54.04	99.01	97.34	DRA: 011937, Experiment: DRX279216	LG	GFF08097	180,587,588	18,239,364,388	45.33	54.67	95.38	92.17	DRA: 011937, Experiment: DRX279222
LK	GFE02901	131,108,822	13,241,991,022	45.08	54.92	99.02	97.37	DRA: 011937, Experiment: DRX279217	LG	GFF03179	154,611,148	15,615,725,948	44.87	55.13	96.16	93.65	DRA: 011937, Experiment: DRX279224
LK	GFE02910	135,559,950	13,691,554,950	45.29	54.71	98.94	97.18	DRA: 011937, Experiment: DRX279218	LG	GFF03183	137,362,464	13,873,608,864	44.87	55.13	96.18	93.69	DRA: 011937, Experiment: DRX279225
LK	GFE02911	135,061,888	13,641,250,688	45.28	54.72	99.03	97.39	DRA: 011937, Experiment: DRX279219	LG	GFF08058	123,067,288	12,429,796,088	45.06	54.94	96.04	93.69	DRA: 011937, Experiment: DRX279226
Total		959,223,704	96,880,594,104	-	-	-	-	-	Total		1,094,773,482	110,572,139,682	-	-	-	-	-
After normalization		182,578,084	-	-	-	-	-	-	After normalization		209,039,560	-	-	-	-	-	-

**Table 5:** Summary of unigenes constructed from short-read transcript data

	*Larix kaempferi*			*Larix gmelinii* var. *japonica*		
	Contigs	Unitranscript	Unigene	Contigs	Unitranscript	Unigene
Number of sequences	912,369	118,141	58,396	1,133,931	72,294	36,972
Total length (bases)	619,249,181	154,148,063	68,361,245	744,563,879	98,330,037	49,327,511
Average (bases)	679	1,305	1,171	657	1,360	1,334
Maximum (bases)	17,703	17,703	17,703	19,838	18,429	18,429
Minimum (bases)	172	172	188	170	180	185
N50 (bases)	1,112	2,311	2,246	1,016	2,374	2,474
G + C%	44.2	41.7	41.8	43.7	41.8	41.7
A	172,604,111	44,911,691	19,905,530	209,250,990	28,589,958	14,364,447
T	172,848,343	44,883,640	19,908,628	209,910,416	28,611,870	14,387,526
G	135,206,494	31,849,992	14,133,013	160,755,371	20,361,148	10,199,216
C	138,590,233	32,502,740	14,414,074	164,647,102	20,767,061	10,376,319

ORF predictions based on the unigenes were conducted using the TransDecoder program for the two larches. A total 27,130 and 20,207 ORFs with lengths ranged from 255–14,493 bp (mean 996 bp) and 255–14,493 bp (mean 1,111 bp) and N50 lengths of 1,341 bp and 1,500 bp were predicted for the Japanese and Kuril larches, respectively (Table 6). In the BUSCO analysis, the completeness of the predicted ORFs was 82.0 and 87.5 complete (%) in the Japanese and Kuril larches, respectively.

**Table 6:** Statistics and completeness of the ORFs predicted from RNA-seq short-reads

	*Larix kaempferi*	*Larix gmelinii* var. *japonica*
Number of ORFs	27,130	20,207
Total length (bp)	27,027,003	22,448,592
Average (bp)	996	1,111
Maximum (bp)	14,493	14,493
Minimum (bp)	255	255
N50 (bp)	1,341	1,500
G + C%	45.0	44.6
A	7,714,114	6,465,651
T	7,152,243	5,973,339
G	6,610,707	5,484,799
C	5,549,939	4,524,803
BUSCO v3 (odb10; 1375)		
Complete (%)	82.0	87.5
Complete and single-copy (%)	79.5	84.7
Complete and duplicated (%)	2.5	2.8
Fragmented (%)	6.7	2.8
Missing (%)	11.3	9.7

### Integration of ORFs obtained by Iso-Seq and RNA-Seq analyses

To examine the entire full-length transcriptome sequences, the ORF sequences (high-quality full-length isoforms) obtained by Iso-Seq analysis were merged with ORFs obtained by RNA-Seq analysis. As a result, 107,687 and 87,539 ORFs were generated for the Japanese and Kuril larches, respectively (Table 7). Finally, the merged ORFs were subjected to cluster analysis using cutoffs of 90% identity and 90% length coverage with CD-HIT-EST. The longest ORF was then selected from each cluster as a representative transcript. As a result, 50,690 and 38,684 ORFs were obtained for Japanese and Kuril larches, respectively. The number of ORFs derived from Iso-Seq and RNA-Seq in Japanese larch were 32,610 (64.3%) and 18,080 (35.7%), respectively, and those in Kuril larch were 27,490 (71.1%) and 11,194 (28.9%), respectively. The total lengths of the merged ORFs were 53.8 and 39.0 Mb, and N50 lengths, GC content and BUSCO completeness values were 1,473 bp and 1,413 bp, 46.4% and 45.1%, and 90.5% and 92.1% in the Japanese and Kuril larches, respectively (Table 7). The obtained Japanese larch ORFs were then mapped to the genome sequence of Japanese larch isolate RF27, which was obtained from the NCBI database (Accession No.: WOXR00000000). As a result, 40,607 ORFs (80.1%) were mapped to 52,341 loci, and of these ORFs, 32,877 were uniquely mapped to specific loci. The 52,054 ORFs were extracted from the mapped regions, and a BUSCO analysis generated completeness values of 73.0% and fragmented values of 13.0%. Based on these findings, it is considered that 86.0% of the ORFs constructed in this study correspond to protein-encoding regions in the Japanese larch genome.

**Table 7:** Statistics and completeness of merged ORFs from Iso-seq and RNA-Seq short-reads

	*Larix kaempferi*		*Larix gmelinii* var. *japonica*	
	Merged ORFs (Isoseq + RNA seq)	Merged ORFs after clustering (Accession No.: ICRN01000001-1050690)	Merged ORFs (Isoseq + RNA seq)	Merged ORFs after clustering (Accession No.: ICRM01000001-1038684)
Number of ORFs	107,687	50,690	87,539	38,684
Total length (bases)	136,244,546	53,804,534	101,368,555	39,053,584
Average (bases)	1,265	1,061	1,158	1,010
Maximum (bases)	14,493	14,493	14,493	14,493
Minimum (bases)	145	145	146	146
N50 (bases)	1,665	1,473	1,539	1,413
G + C%	44.8	46.4	44.5	45.1
A	39,163,057	15,014,796	29,209,051	11,107,507
T	35,977,224	13,798,508	27,009,101	10,313,639
G	33,156,639	13,227,788	24,804,297	9,554,898
C	27,947,626	11,763,442	20,346,106	8,077,540
Category of ANGEL				
Confident	-	14,264	-	120,23
Confident- complete	-	8,933	-	8,315
Confident-5' partial	-	5,174	-	3,589
Confident-3' partial	-	122	-	105
Confident- internal	-	35	-	14
Likely-NA	-	4,143	-	3,142
Suspicious-NA	-	6,991	-	6,611
Dumb-complete	-	7,126	-	5,644
Dumb-5'partial	-	6	-	3
Dumb-3'partial	-	80	-	67
Category of Transdecorder				
Complete	-	10,099	-	6,489
5' Prime_partial	-	2,946	-	1,552
3' Prime_partial	-	1,885	-	1,023
Internal	-	3,150	-	2,130
BUSCO v3 (odb10; 1375)				
Complete (%)	90.6	90.5	92.1	92.1
Complete and single-copy (%)	39.8	83.3	32.8	84.8
Complete and duplicated (%)	50.8	7.2	59.3	7.3
Fragmented (%)	3.1	3	1.5	1.5
Missing (%)	6.3	6.5	6.4	6.0

### Comparison of ORFs between Japanese and Kuril larches

**Fig. 1:**
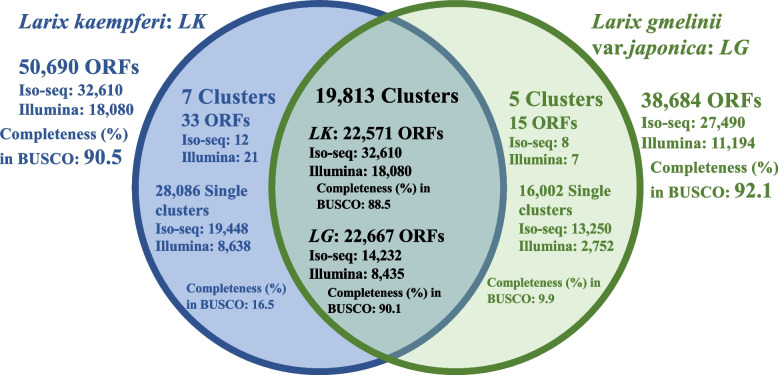
Venn diagram showing the overlap between open reading frames obtained from Japanese larch and Kuril larch in this study

To clarify the interspecific relationship between the Japanese and Kuril larches, we compared 50,690 and 38,684 ORFs from the two species using OrthoFinder (Fig. 1), and searched for sequences in the NCBI’s non-redundant protein (NR) database using DIAMOND software with the more sensitive mode. The results are shown in the Venn diagram in Fig. 1 and are summarized in Table 8. A total of 19,813 clusters, comprising of 22,571 Japanese larch ORFs and 22,667 Kuril larch ORFs, were contained in the intersection of the Venn diagram. The BUSCO completeness values for the ORFs from the Japanese and Kuril larches were 88.5% and 90.1%, respectively. Among these ORFs, the number of ORFs with significant (E-value ≤ 1e-10) matches against plant entries in GenBank nucleotide divisions (gbpln) in the NR database was 20,335 (90.1% of all ORFs) and 20,464 (90.3% of all ORFs) for Japanese and Kuril larches, respectively. A total of 28,119 ORFs (including seven clusters, which consisted of 33ORFs) were unique to Japanese larch, with a BUSCO completeness of 16.5% and 18,919 ORFs matches against gbpln. For the Kuril larch, a total of 16,017 ORFs (including five clusters, which consisted of 15 ORFs) were species-specific, with a BUSCO completeness of 9.9% and 11,534 ORFs matches against gbpln. In the intersection of the Venn diagram, approximately 90% of ORFs from the two species were annotated in gbpln (Additional File 1). However, in the species-specific region of the Venn diagram, the proportion of “no hits” against the NR database was relatively high, and the proportion of gbpln “hits” was relatively low (Additional File 1). To assign functional characteristics to the ORFs from the two species, a gene ontology (GO) analysis was performed. After classifying the ORFs into the GO categories (BP: biological process, CC: cellular component, MF: molecular function), the distribution of the GO terms was found to be similar between the two species (Additional File 2).

**Table 8:** Interspecific comparison of Japanese larch and Kuril larch ORFs

	Intersection of Venn diagram		Specific to *Larix kaempferi*	Specific to *Larix gmelinii* var. *japonica*
Number of clusters	19,813		7	5
	*Larix kaempferi*	*Larix gmelinii* var. *japonica*		
Number of ORFs				
PacBio Iso-seq	13,150	14,232	12	8
Illumina RNA-seq	9,421	8,435	21	7
Total	22,571	22,667	33	15
Single clusters				
PacBio Iso-seq	-	-	19,448	13,250
Illumina RNA-seq	-	-	8,638	2,752
Total	-	-	28,086	16,002
Homologs to gbpln in NCBI				
PacBio Iso-seq	11,908	12,917	13,098	9,613
Illumina RNA-seq	8,427	7,547	5,821	1,921
Total	20,335	20,464	18,919	11,534
No hits against NR database				
PacBio Iso-seq	932	1,052	5,057	3,354
Illumina RNA-seq	743	665	2,280	759
Total	1,675	1,717	7,337	4,113
BUSCO v3 (odb10; 1,375)				
Complete (%)	88.5	90.1	16.5	9.9
Complete and single-copy (%)	84.6	84.9	14.5	9.2
Complete and duplicated (%)	3.9	5.2	2.0	0.7
Fragmented (%)	3	1.7	13.1	13.5
Missing (%)	8.5	8.2	70.4	76.6

### Comparison of ORFs against other species

The ORFs were searched against the NR database using the DIAMOND program with more sensitive mode (Additional File 3, 4). For the Japanese and Kuril larches, a total of 39,661 ORFs (78.2% of all ORFs) and 31,336 ORFs (81.0% of all ORFs) had significant BLAST matches (E-value ≤ 1e-10), respectively (Additional File 3, 4, 5). Similarities between the 50,690 and 38,684 ORFs from Japanese and Kuril larches were searched by BLASTP with an E-value cutoff of 1e-10, respectively. The finding showed that 41,301 ORFs (81.5% of all ORFs) from Japanese larch had significant BLAST matches with the ORFs from Kuril larch (Additional File 3, 4, 5). Conversely, 34,933 ORFs (90.3% of all ORFs) from Kuril larch were matched to ORFs from Japanese larch (Additional File 3, 4, 5). When we compared the protein sequence similarities of the genes among *A. sachalinensis*, *P. lambertiana*, *Populus trichocarpa*, *Arabidopsis thaliana*, and *C. japonica* using a BLASTP searches with an E-value cutoff of 1e-10 (Additional File 3, 4, 5), the number ORFs shared by each of these species with Japanese and Kuril larches was 38,513 and 32,794 for *Abies*; 36,156 and 30,821 for *Pinus*; 36,149 and 30,739 for *Cryptomeria*; 32,820 and 27,978 for *Populus*; 32,351 and 27,572 for *Arabidopsis*, respectively.

### Phylogenetic analysis of flowering-related genes

**Fig. 2:**
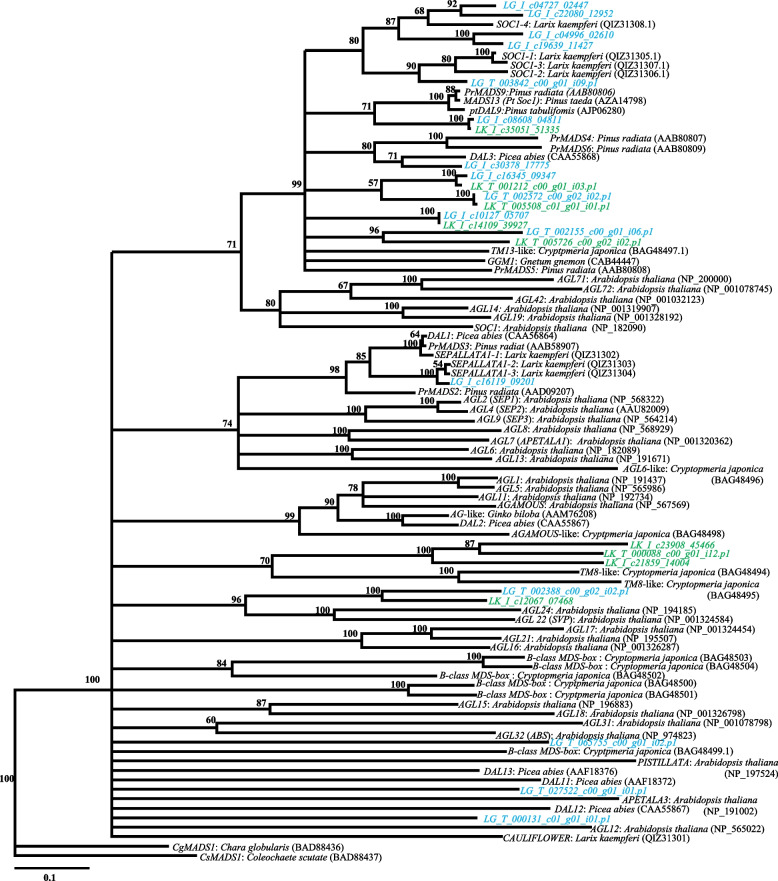
Phylogenetic tree showing the relationships between known MADS-box genes and a set of other angiosperm and gymnosperm sequences. Japanese larch open reading frames are shown in green. Kuril larch open reading frames are shown in blue. Numbers adjacent to some nodes show bootstrap percentages

**Fig. 3:**
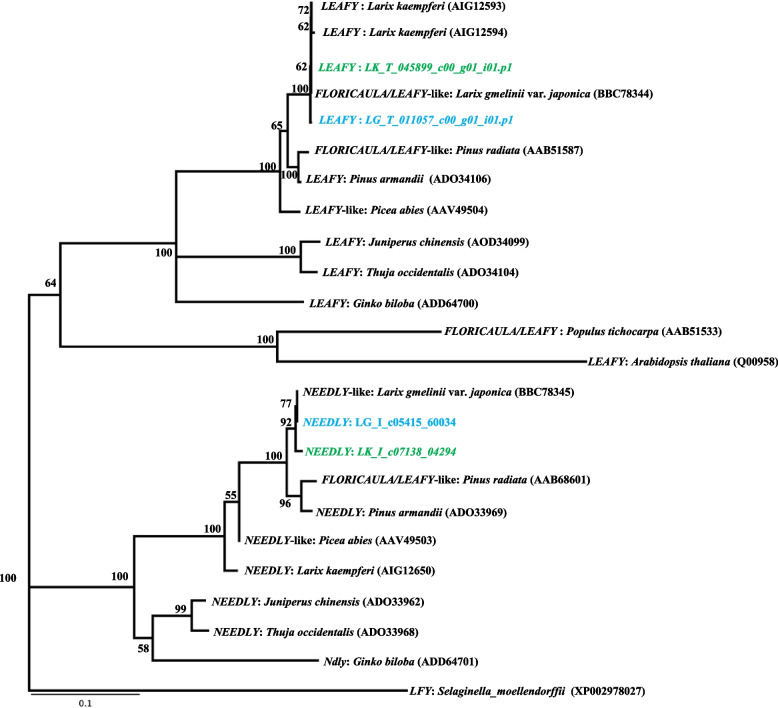
Phylogenetic tree showing the relationships between known *LEAFY* and *NEEDLY* genes and a set of other angiosperm and gymnosperm sequences. Japanese larch open reading frames are shown in green. Kuril larch open reading frames are shown in blue. Numbers adjacent to some nodes show bootstrap percentages

**Fig. 4:**
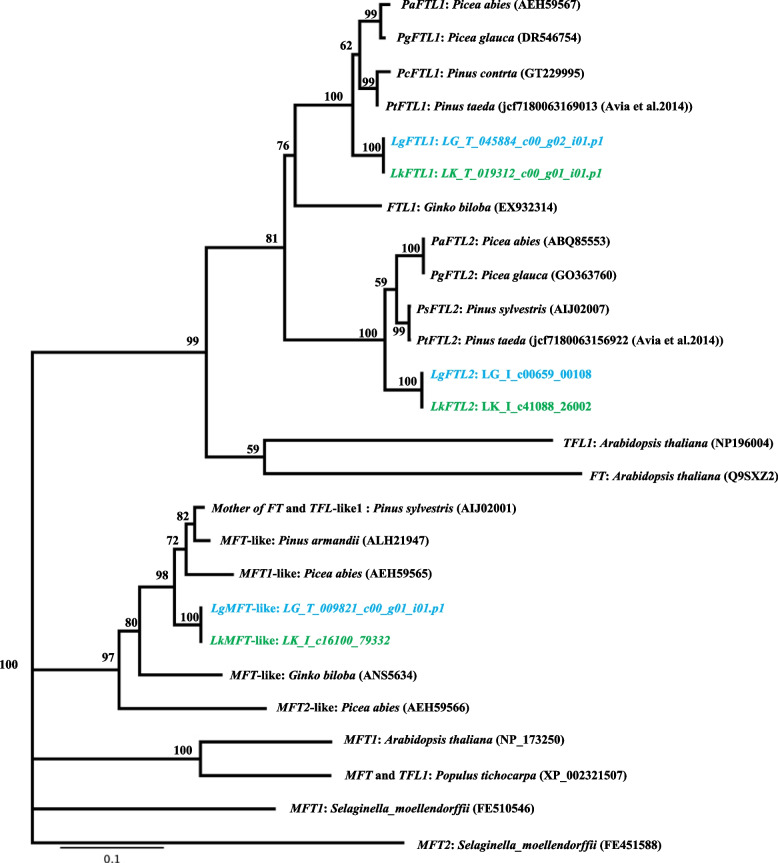
Phylogenetic tree showing the relationships between known *FT/FT*-like and *MFT* genes and a set of other angiosperm and gymnosperm sequences. Japanese larch open reading frames are shown in green. Kuril larch open reading frames are shown in blue. Numbers adjacent to some nodes show bootstrap percentages

**Fig. 5:**
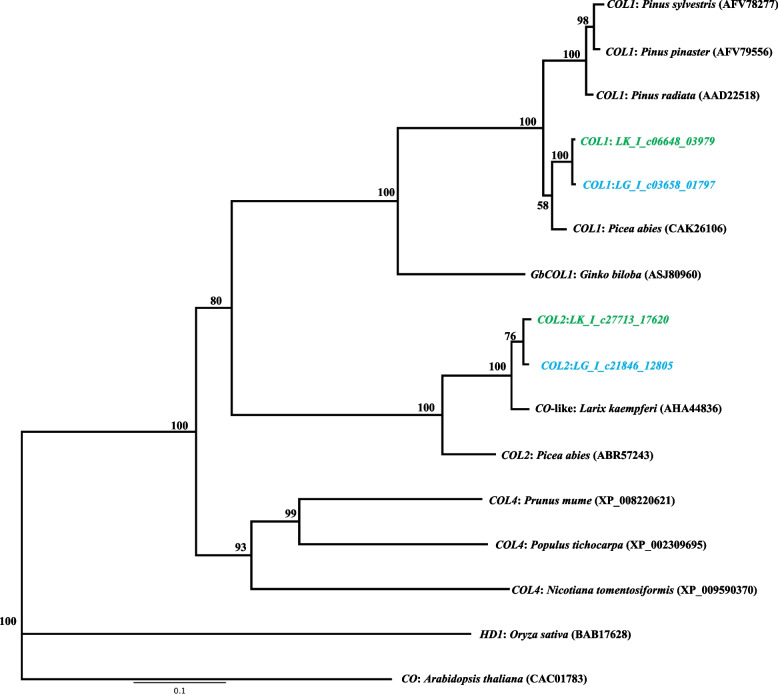
Phylogenetic tree showing the relationships between known *CONSTANS* genes and a set of other angiosperm and gymnosperm sequences. Japanese larch open reading frames are shown in green. Kuril larch open reading frames are shown in blue. Numbers adjacent to some nodes show bootstrap percentages

Twenty-five candidate ORFs that were similar to the Type II MADS-box gene were founded in Japanese (9 ORFs) and Kuril larches (16 ORFs). A phylogenetic tree was constructed together with previously identified MADS-box genes sequence from *Larix*, *Pinus*, *Picea*, *Cryptomeria*, *Gnetum*, *Arabidopsis*, *Coleochaete*, and *Chara* (Fig. 2, Additional File 6). Among the 25 *Larix* ORFs identified in this study, 16 were shared by the members of a subgroup in the *Soc1* clade (TM3 clade) including *Pinus* and *Picea* sequences. Among the 16 *Larix* ORFs, five sequences (LG_I_c04727_02447, LG_I_c22080_12952, LG_I_c04996_02610, LG_I_c19639_11427, and LG_T_003842_c00_g01_i09.p1) were similar to the previously reported sequences in the Japanese larch, but the other 11 sequences were located in different clades and would thus be novel candidate TM3-like genes. The four ORFs were annotated as the *LEAFY/NEEDLY* gene by the BLASTP searches. The amino acid sequences were identical in the two larch species, and similar to those previously reported for Japanese and Kuril larches. In the phylogenetic tree, the detected *LEAFY/NEEDLY* genes were similar to the orthologous sequences in *Pinus* and *Picea* species, which belonged to the same clade (Fig. 3, Additional File 7). Based on the BLASTP searches, three ORFs that were similar to *FLOWERING Locus T-*like genes/*Mother of FLOWERING Locus T-*like *(MFT-*like*)* genes were annotated in both of the larches. In the phylogenic tree, four out of six of the *Larix* ORFs were assigned to the clades *FTL1* and *FTL2*. Each of the *Larix FTL1* and *FTL2* sequences were accompanied by orthologous sequences of *Pinus* and *Picea* species within the clade (Fig. 4, Additional File 8). As for *MFT-*like genes, two amino acid sequences (LK_I_c16100_79332 and LG_T_009821_c00_g01_i01.p1) were identical between the two larch species and the sequences were grouped in the same cluster as *Pinus_MFT, Picea_MFT1* in the phylogenetic tree (Fig. 4, Additional File 8). Four ORFs of *CONSTANCE (CO)-like* genes were annotated from the BLASTP searches. Two of the four ORFs annotated as *CO* genes were located in the coniferous tree *COL1* gene clade. The other two ORFs were located in the *COL2* gene clade with the *Picea_COL2* in the phylogenetic tree (Fig. 5, Additional File 9). These results showed that the Japanese larch ORFs were similar to the previously reported sequences in the Japanese larch, and that the Kuril larch ORFs contained novel candidate genes.

## Discussion

### Full-length (FL) transcriptome collection

In this study, we successfully sequenced 107,687 and 87,539 transcripts (Iso-Seq + RNA-seq) derived from total-RNA collected from the shoots, cambium, and needles of Japanese and Kuril larches, respectively (Table 7). To remove any redundancy in the unigenes sequences derived from alternative splicing or heterozygosity in the Japanese and Kuril larches, the ORFs were clustered using a minimum identity of 90% and a high coverage of at least 90% using CD-HIT-EST. Based on these sequences, a total of 50,690 and 38,684 ORFs were predicted for Japanese and Kuril larches, respectively. The number of the ORFs obtained by Iso-Seq was approximately three-fold that obtained by RNA-Seq; however, the gene coverages estimated by BUSCO for Iso-Seq data was lower than that estimated for RNA-Seq data (Table 2,6). These results could be attributed to the following factors. First, since the Iso-Seq data are derived from long-reads, they reflect full-length sequences, which enables more accurate ORF estimation, and can also provide isoform information; however Iso-Seq are less comprehensive than a short-read data. Second, while short-read data have a relatively higher gene coverage than Iso-Seq data, primarily due to the large amount of sequence data obtained, instances of miss-assembly during the assembly process can reduce the accuracy of ORF prediction from unigenes [37]. Indeed, the likelihood of mis-assembly is particularly high in genomes of coniferous trees because the genomes are large, complex and have numerous repetitive sequences [42, 43]. Therefore, we attempted to construct the ORFs using the Iso-Seq and RNA-Seq reads by a clustering approach. As a result, we constructed unigenes including approximately 90% of the single core genes defined by BUSCO analyses in both the Japanese and Kuril larches.

Transcript sequencing has been reported for various coniferous species, and the number of unigenes has varied among species. For example, *C. japonica* has 34,731 unigenes [23]; *P. bungeana* has 88,092 unigenes [32]; *A. sachalinensis* has 158,542 transcripts [33]; *P. taeda* has 50,172 gene models (15,653 high confidence) [44]; *P. abies* has 28,345 high-confidence genes [14]; *P. menziesii* var. menziesii has 22,257 high-quality full-gene models [17]; *P. lambertiana* has 85,053 gene models (13,936 high confidence) [16]; *P. tabuliformis* has 80,495 genes [18]; *S. sempervirens* has 118,906,495 genes [20] and *Larix kaempferi* has 299,637 assembled transcripts [36], 85,446 unigenes [45]. In this study, the number of ORFs predicted using Iso-Seq and RNA-Seq data was comparable to values reported in these previous studies, especially when the sequences obtained in this study are compared with previously published Japanese larch data; for example, the assembly statistics were similar except that the number of ORFs was approximately 1/6 this study compare to previous study [36]. The BUSCO completeness values estimated in this study were higher than those for the *P. lambertiana* and *P. taeda* genomes [16]. These findings indicate that the approach used to construct the unigenes by merging Iso-Seq and RNA-Seq sequences was very effective for developing a comprehensive and extensive collection of full-length transcriptome sequences.

Comparing the ORFs collected from the two larch species, 19,813 clusters comprising 22,517 and 22,667 ORFs for Japanese and Kuril larches were located in the intersection of the Venn diagram shown in Fig. 1. Only seven clusters were unique to Japanese larch and five to Kuril larch; these clusters contained 33 and 15 ORFs, respectively, and 28,086 and 16,002 ORFs were not clustered, respectively. The BUSCO completeness for the common ORFs among Japanese and Kuril larches was 88.5% and 90.1%, respectively, whereas that for the species-specific ORFs was 16.5% and 9.9%, respectively (Fig. 1). These findings suggest that highly comprehensive and reliable ORFs were found in the intersection of the Venn diagram. Conversely, in the specific regions of the Venn diagram (i.e., the species-specific regions to the sides of the intersection) may contain species-specific sequences, and the possibility of contamination cannot be unfortunately, completely ruled out. In addition, the proportion of ORFs in this region that could not be annotated is relatively high, and while these ORFs may be novel sequences or long non-coding RNAs, they are among the factors that decrease the BUSCO score (Fig. 1). The ORFs that we obtained and characterized provide a valuable resource for a molecular breeding and comparative study of functional genetics in coniferous species.

### Phylogenetic analysis of flowering signal genes

In *A. thaliana*, flowering control signals are induced by multiple external environmental and internal physiological factors, such as long days, autonomous vernalization, age, and gibberellins, and multiple control pathways are involved in transmitting changes in these factors [46]. Moreover, several key transcription factor genes, such as *SOC1*, *LFY*, *FT* and *CO*, that integrate multiple control pathways based on these environmental factors have been identified [46]. In *Larix* species, there is considerable year-to-year variation in flower production and, few consecutive flowering events are typically observed [41]; indeed, this is one of the problems that needs to be addressed in breeding. In this study, the key transcription factor genes, *SOC1*, *FT*, and *LFY*, and *CO* candidate ORFs involved in the regulation of flowering onset in Japanese and Kuril larches were screened from the collected ORFs using *A. thaliana* annotation information, and a phylogenetic tree was generated using similar genes found in other coniferous tree species.

When *SOC1* is induced at the top of the shoot, *SOC1*, along with *AGL24*, directly activate the flower meristem identity gene *LFY* in *A. thaliana* [44]. *SOC1*-like genes have been isolated from several gymnosperms (e.g., *G. gnemon* [47], *P. abies* [48, 49], *P. radiata* [50], and *L. kaempferi* [51]). Further, gene expression and transgenic studies of *SOC1-*like genes isolated from Japanese cedar revealed that these genes play important roles in development of male and female strobili [52]. However, in Japanese larch and *P. tabuliformis*, the expression of some *SOC1* homologs was reported to increase with age, implying that *SOC1* plays a different role in *Arabidopsis* [51, 53, 54]. In the phylogenetic tree generated in this study, many Japanese and Kuril larch ORFs were located in polyphyletic clades of the *TM3*-related gene group to which *SOC1* of *Arabidopsis* and *AGL24*-like ORFs from the two larch species were grouped together with the outgroup of *AGL24* in *A. thaliana* (Fig. 2); however, both ORFs are reflected the phylogenetic relationships among these species (Fig. 2). Additionally, in this study, the *SOC1* homologs of Kuril larch were more similar to the Japanese larch *SOC1* homolog previously reported than the one obtained from Japanese larch in this study. Presently, reasonable explanations were unfortunately unable to be found. This could be clarified by some verifications (for example, comparison of gene expression patterns of each homolog) in the future.

In angiosperms, *LEAFY/FLORICAULA* (*LFY/FLO*) is regulated directly by *SOC1*, which regulates downstream *MADS-box B-Clas*s and *C-class* genes involved in floral meristem determinacy [46, 55, 56]. All major groups of existing gymnosperms are known to carry two paralogous *LFY*-like genes (*LFY/NEEDLY*) [57–59] which contribute directly to reproductive structure formation [60]. In *P. abies*, it has been clarified that *NEEDLY* is a potential mediator in the transition from vegetative shoots to female cones [61]. In *P. taeda*, the *NEEDLY* gene encodes a functional ortholog of the *FLORICAULA/LEAFY* genes of angiosperms [62]. In this study, the ORFs were screened in the two larch species, and the sequences were almost identical (Fig. 3, Additional file 7). The *LFY*-like genes in the genus *Larix* reflected the phylogenetic relationships of both *LFY*-like and *NEEDLY* genes, which is consistent with previously reported results [59].

*FLOWERING LOCUS T* (*FT*) which belongs to the *CENTRORADIALIS/TERMINAL FLOWER 1/SELF-PRUNING* (*CETS*) gene family, is an important floral integrator that is induced by long-distance signals that contributes to the activation of the meristem identity gene class A (*AP1:APETALA1*) in *Arabidopsis* [63–65]. In *P. abies*, genes of the *CET* gene family were isolated (*MOTHER OF FT AND TFL1* (*MFT*)-like clade, *PaMFT1* and *PaMFT2*, and *FT* and *TERMINAL FLOWER 1*(*TFL1*) clades, *PaFTL1* and *PaFTL2*); when overexpressed in *A. thaliana*, the *PaFTL1* and *PaFTL2* genes suppress flowering, but *PaMFT1* and *PaMFT2* have no effect [66]. In particular, *PaFTL2* has been shown to control growth cessation and bud set in response to short day (SD) length, and bud burst in response to elevated temperatures [67, 68]. A *Pinus sylvestris* homolog to *PsFTL2* employs a similar mechanism for regulating the timing of growth cessation in conifers [69]. In the phylogenetic tree estimated in the present study, the ORFs related to *FTL1, FTL2* and *MFT* were screened in the two larch species. The amino acid sequences for each ORF were the same and were located in the *Picea* and *Pinus* subgroup on the phylogenetic tree (Fig. 4, Additional file 8). Therefore, the *FTL2* genes in genus *Larix* may have functions that are similar to those reported previously [67–69].

*CONSTANS* (*CO*) is involved in the photoperiod flowering pathway [70]. Specifically, in *Arabidopsis*, *CO* senses the photoperiod and integrates the circadian clock and light signals to induce downstream photoperiod-specific *FT* transcription [71]. In gymnosperms, a *CO*-like gene has been isolated and identified in several species (*P. sylvestris*, AFV78277.1, *P. radiata*, AAD22518.1, *P abies*, [68, 70], *L. kaempferi*: AHA4436). In *P. abies*, two *CO-*like genes, *PaCOL1* and *PaCOL2*, were isolated, and transcription levels of these genes in shoots and needles were significantly reduced under SD prior to growth cessation and bud formation, suggesting their involvement in the photoperiodic control of shoot elongation [72]. In the phylogenetic tree generated in the present study, the ORFs that were similar to the *CO*-like gene in the genus *Larix* accurately reflected the phylogenetic relationships of these species and formed a subgroup with the genus *Picea* (Fig. 5). In both of the larches, the obtained *COL2* ORFs were similar to a previously registered sequence (AHA44836) (Fig. 5, Additional file 9). Since larches are deciduous, the detection of SD prior to growth cessation is important, and a clear SD response mechanism may exist in larch species.

However, further research is needed to identify and clarify the role of screened flowering signal-related ORFs in Japanese and Kuril larches, and information about the ORFs could be used to elucidate flowering mechanisms and to achieve stable flower production in future studies.

## Conclusion

In this study, we obtained 50,690 and 38,684 ORFs from cambium, needle, and shoot samples of Japanese and Kuril larches. By collecting ORFs using Iso-Seq and RNA-Seq, we constructed comprehensive reference genes for the two species. These genes were supported by high BUSCO completeness (90.5% in Japanese larch and 92.1% in Kuril larch). Our interspecific comparison revealed that the shared sequences were generally highly comprehensive and comprised of reliable ORFs. Comparisons with previously published larch genome sequences showed that the sequence collection of Japanese larch genes was comprehensive and increased our knowledge of the larch genome. The flowering signal-related ORFs were screened from the obtained ORF sequences of the two species. Furthermore, comparisons with closely related species revealed that the sequence of the flowering signal-related genes showed higher homologies to previously identified sequences from coniferous trees, indicating that the amino acid sequences of important domains are well conserved depending on their phylogenetic relationships. The isoform and RNA-Seq short-read data obtained in this study would also be useful for detecting gene loci and constructing gene models for larch genome sequences. In addition, the obtained reference sequences will provide a reference for the molecular breeding of the two larch species, and for future conifer genome evolutionary and functional genomics research.

## Methods

### Plant material

All of the plant materials used in this study were shown in Additional File 10. Those materials were of breeding material (plus-tree clone) or genetic resources genus Larix in Japan, and clonally propagated by grafting. The differences in age of the trees shown in Additional File 10 indicate differences in the number of years elapsed since the original trees were propagated by grafting. All of the materials employed in this study are preserved as clonally propagated trees at either the Tohoku or Hokkaido Regional Breeding Office, Forest Tree Breeding Center, Forestry and Forest Products Research Institute, Forest Research and Management Organization in Iwate or Hokkaido prefectures, Japan. For the sampling method, we sampled from those grafted trees to collect as many genes as possible from various organs at different timings for the comprehensiveness of the gene collection. The timing of sampling and each organs are shown in Additional File 10.

### Full-length (FL) isoform sequencing

To construct the EST libraries, we sampled the branches, cambium region, and needles throughout the annual season of a 56-year-old Japanese larch plus-tree clone from Takizawa, Iwate Prefecture (Additional File 10). Cambium tissue samples were collected from the trunk at breast height (approximately 1.2–1.3-m height) and branches were randomly sampled from the tree crown. Needle samples were collected from short shoots on the branches. The same tissues were collected from a 57-year-old Kuril larch plus-tree clone from Ebetsu, Hokkaido Prefecture (Additional File 10). Total RNA was isolated using an RNeasy Plant Mini kit (QIAGEN, Gaithersburg, MD, USA) and Maxwell® RSC plant RNA kit (Promega, Madison, WI, USA). The amount and quality of total RNA were assessed using a NanoDrop 2000 (Thermo Scientific, USA) and Agilent Bioanalyzer 2100 system (Agilent Technologies, Palo Alto, CA, USA). Only high-quality total RNA extracts with RNA integrity numbers (RINs) > 7.0 were selected for analysis and extracted RNA from each organ was bulked for RNA library construction. The sequencing libraries were then prepared for isoform sequencing (Iso-Seq™) using a Clontech SMARTer PCR cDNA Synthesis Kit and a BluePippin™ Size-Selection System (Sage Science, Inc., Beverly, MA, USA) according to the manufacturer’s instructions. Briefly, the cDNA for the library was constructed as per the Clontech SMARTer-PCR cDNA Synthesis Sample Preparation Guide. Libraries of 1–2 kb, 2–3 kb, 3–6 kb, and 5–10 kb from Japanese larch and 1–2 kb, 2–3 kb, 3–4 kb, and 4–10 kb from Kuril larch were selected using the BluePippin™ Size-Selection System (Sage Science, Inc.), purified, and end-repaired before the blunt-end SMRTbell adapters were ligated. The libraries were quantified using Quant-IT PicoGreen (Invitrogen, Waltham, MA, USA) and qualified using the Agilent Technologies 2100 Bioanalyzer (Agilent Technologies, USA). Subsequent sequencing was performed in 9 and 10 SMRT Cells using P6C4 in PacBio RSII for Japanese and Kuril larches, respectively. In the obtained ROIs, full non-chimeric ROIs were clustered using the ICE software package and polished with non-full, non-chimeric ROIs using the Quiver software package [73]. Using CD-HIT-EST v4.6.5 [74], the polished full non-chimeric ROIs were clustered to build collapsed redundant sequences (Additional File 11).

### Construction of unigenes by short-read sequences

Cambium, needles, and branch tissues were sampled from seven trees each of Japanese and Kuril larches for RNA sequencing (Additional File 10). To collect gene sequences involved in flowering, the branch samples of GFE32200, GFE32203 (in Japanese larch), and GFF08127 (in Kuril larch) during the flower bud differentiation period, namely from June to September, were included [75, 76]. It had previously been observed that the three clones produced flowers in consecutive years. Therefore, the branch samples of these clones were thought to contain flower buds in an early development stage.

Total RNA extraction and assessments of RNA amount and quality were performed by the same procedures described above for the isoform sequences. Only RNA with RIN > 7.0 from each tree was used and extracted RNA from each organ was bulked for library construction. Using a TruSeq RNA Sample Prep kit (Illumina, Inc., San Diego, CA, USA), cDNA synthesis from the bulked RNA samples from each tree, nebulization, adaptor ligation (including index tagging for individual recognition), bridge PCR, and 101 bp paired-end sequencing were performed on Illumina HiSeq 2500/4000 platforms. The quality and adaptor trimming were performed by PRINSEQ v0.20.4 [77] and FastX_clipper in the FASTX toolkit (http://hannonlab.cshl.edu/fastx_toolkit/), and the resultant reads were used for de novo assembly by Trinity v2.8.5 [78]. The trimmed reads were then mapped against the contigs using Bowtie2 v2.3.5.1 [79], and the contigs with FPKM > 1 were filtered as “unitranscripts” to select intrinsic genes and to exclude possible contaminants. Finally, the longest unitranscripts were selected from the splicing variants in the unitranscript and defined as unigenes (Additional File 11). In the unigene assembled by short-read sequencing (RNA-Seq), the potential coding region and corresponding encoded proteins of Larix genes were identified and retrieved using TransDecoder v5.5.0 (https://github.com/TransDecoder/TransDecoder).

### ORF prediction from Iso-Seq sequences

The full-length ORFs were predicted from the high-quality, non-redundant Iso-Seq sequences using the ANGEL software (https:/gthub.com/PacificBiosciences/ANGEL). Briefly, a training dataset was created using outputs from the longest ORF in all frames (Dumb ORF prediction) and training was performed using a coding potential classifier based on the obtained training data (ANGEL classifier training). Then, using this training data, ORF prediction was performed from the Iso-Seq data. The data generated by the ANGEL software were classified according to nine criteria: (i) confident-complete, where a full-length (FL) single-ORF was generated; (ii) confident-5’ partial, where a single-ORF was generated without a 3’ terminal sequence; (iii) confident-3’ partial, where a single-ORF was generated without a 5’ terminal sequence; (iv) confident-internal, where a single-ORF was generated without a start and stop codon; (v) likely-NA, where multi-ORFs were generated, and the length of only one ORF was above the threshold; (vi) suspicious-NA, where multi-ORFs were generated, and the length of some ORFs were above the threshold; (vii) dumb-complete, where a training set was generated with FL ORFs; (viii) dumb-5’ partial, where a training set was generated without a 3’ terminal sequence; (ix) dumb-3’ partial, where a training set was generated without a 5’ terminal sequence.

### Consensus transcriptome construction and characterization

To construct the consensus transcriptome, the ORF sequences predicted by ANGEL and the ORFs predicted from the unigenes derived from the RNA-Seq were clustered using CD-HIT-EST v4.6.5 [74] (Additional File 11). A metric of gene completeness for these assemblies was estimated using BUSCO v3.0 [80] (https://busco.ezlab.org/) with the embryophyta odb10 dataset. The resulting Japanese larch ORF sequences were mapped to a published genomic sequence of Japanese larch isolate RF27 (Accession No.: WOXR00000000) using GMAP v2020.06.01 [81].

### ORFs comparison between two larches

We used OrthoFinder v2.2.3 [82, 83] to identify orthologous and species-specific genes for the ORFs between Japanese and Kuril larches using default parameters.

### Functional annotation of the ORFs

The ORFs were compared against the NCBI non-redundant protein sequence (NR) database (ftp://ftp.ncbi.nih.gov/blast/db/FASTA/nr.gz) using DIAMOND software [84] with the more sensitive mode. The similarities among *Larix kaempferi* and *Larix gmelinii* var. *japonica* were compared using BLASTP. Similarities to the protein sequences of *Arabidopsis thaliana* (Araport11 201606pep, https://www.araport.org), *Abies sachalinensis* (TodoFirGenes [http://plantomics.mind.meiji.ac.jp/todomatsu/]), *Pinus lambertiana* (TreeGenes PILA.1_5 peptides), *Populus trichocarpa* (Ensembl v4.1 proteins), and *Cryptomeria japonica* (MSSID: IABU01000001-01034731) were identified using BLASTP (Additional File 3, 4) with an E-value cutoff of 1e-10. The similarity search against the NR database (ftp://ftp.ncbi.nih.gov/blast/db/FASTA/nr.gz) in xml format was applied to GO assignments by BLAST2GO v4.0 [85]. The ORFs annotated to MADS-box, *CO*, *FLO/LFY*-like, and *FTL/MFT* genes in Japanese and Kuril larches were aligned against those of the other plant species obtained from NCBI’s GenBank using the ClustalW module implemented in the Geneious software program [86] (https://www.geneious.com) with the sequences of other species retrieved from NCBI’s GenBank. The phylogenetic relationships among these genes were inferred using the neighbor-joining method with bootstrap analysis (10,000 replicates) to assess the support for each branch.

## Supplementary Information

**Additional file 1. **Stacked percent of the ORFs with top hits against NCBI nr database in each region on the Venn diagram. Blue shows the plant and fungal species (gbpln) in the GenBank nucleotide divisions. Orange shows ratio of “no hit”. Gray showed ratio of other divisions. LK and LG shows Japanese and Kuril larch, respectively.

**Additional file 2. **Gene ontology (GO) categories in biological process (BP) that encoded proteins with sequence similarity (E-value ≤ 1e-10) in the NCBI database. a) Japanese larch, b) Kuril larch. Gene ontology (GO) categories in cellular component (CC) that encoded proteins with sequence similarity (E-value ≤ 1e-10) in the NCBI database. a) Japanese larch, b) Kuril larch. Gene ontology (GO) categories in molecular function (MF) that encoded proteins with sequence similarity (E-value ≤ 1e-10) in the NCBI database. a) Japanese larch, b) Kuril larch.

**Additional file 3. **Description of Japanese larch used in this study.

**Additional file 4. **Description of Kuril larch used in this study.

**Additional file 5. **Number of open reading frames (ORFs) similar to NCBI sequeces and other species according to BLASTp E-value cutoff values. a) ORFs obtained from Japanese larch, b) ORFs obtained from ORF Kuril larch.

**Additional file 6. **Alignment of known MADS-box genes and a set of other angiosperm and gymnosperm sequences. Japanese larch open reading frames are shown in green. Kuril larch open reading frames are shown in blue.

**Additional file 7. **Alignment of known LEAFY and NEEDLY genes and a set of other angiosperm and gymnosperm sequences. Japanese larch open reading frames are shown in green. Kuril larch open reading frames are shown in blue.

**Additional file 8. **Alignment of known FT/FT-like and MFT genes and a set of other angiosperm and gymnosperm sequences. Japanese larch open reading frames are shown in green. Kuril larch open reading frames are shown in blue.

**Additional file 9. **Alignment of known CONSTANS genes and a set of other angiosperm and gymnosperm sequences. Japanese larch open reading frames are shown in green. Kuril larch open reading frames are shown in blue.

**Additional file 10. **List of samples collected in this study.

**Additional file 11. **Summary of assembly and characterization.

## Data Availability

The datasets generated in this study are available at the INSDC (DDBJ, EMBL, and GenBank) under the BioProject, PRJDB11621 (Iso-Seq and RNA-Seq; DRA011937, ORFs; ICRN01000001-01,050,690 (Japanese larch) and ICRM01000001-01,038,684 (Kuril larch)) (https://www.ncbi.nlm.nih.gov/bioproject/PRJDB11621, https://www.ncbi.nlm.nih.gov/nuccore/). The protein sequences of MADS-box, *LEAFY/NEEDLY*, *FT/FT*-like, *MFT*, and *CONSTANS* applied to the phylogenetic analyses were downloaded from the protein database in NCBI (https://www.ncbi.nlm.nih.gov/protein/). All of the materials employed in this study are preserved in the Tohoku and Hokkaido Regional Breeding Office, Forest Tree Breeding Center, Forestry and Forest Products Research Institute, Forest Research and Management Organization in Iwate and Hokkaido prefectures, Japan.

## Abbreviations

MF: Molecular function
NCBI: National Center for Biotechnology Information
ORF: Open reading frames
ROI: Read of inserts
