# Supplementary material for: Comprehensive collection of genes and comparative analysis of full-length transcriptome sequences from Japanese larch (Larix kaempferi) and Kuril larch (Larix gmelinii var. japonica)
Source: BMC Plant Biol. 2022 Oct 4;22:470. doi: 10.1186/s12870-022-03862-9 (PMC9531402; doi:10.1186/s12870-022-03862-9)
Supplement: Supplementary file 1 — Additional file 1. Stacked percent of the ORFs with top hits against NCBI nr database in each region on the Venn diagram. Blue shows the plant and fungal species (gbpln) in the GenBank nucleotide divisions. Orange shows ratio of “no hit”. Gray showed ratio of other divisions. LK and LG shows Japanese and Kuril larch, respectively. [file 12870_2022_3862_MOESM1_ESM.pdf]

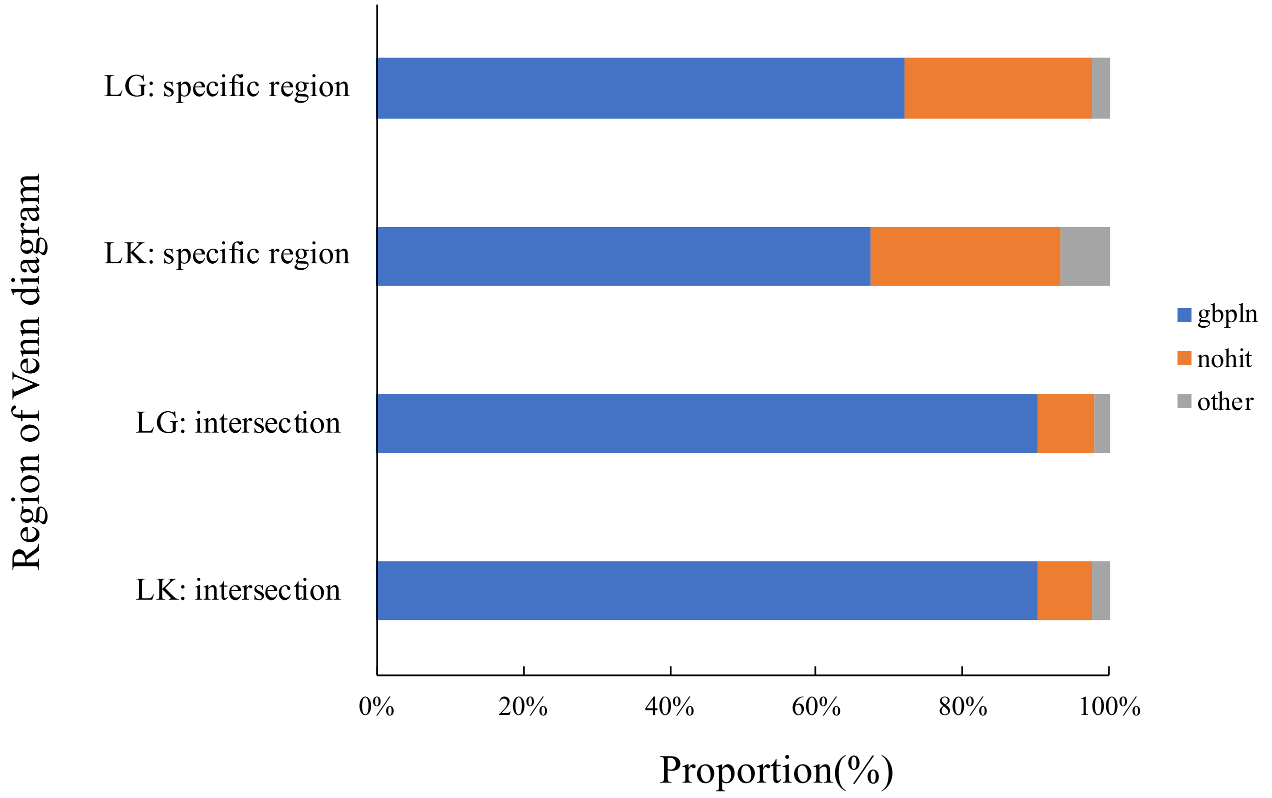

Additional File 1 Stacked percent of the ORFs with top hits against NCBI nr database in each region on the Venn diagram. Blue shows the plant and fungal species (gbpln) in the GenBank nucleotide divisions. Orange shows ratio of "no hit". Gray showed ratio of other divisions. LK and LG shows Japanese and Kuril larch, respectively.
