# Supplementary material for: Comprehensive collection of genes and comparative analysis of full-length transcriptome sequences from Japanese larch (Larix kaempferi) and Kuril larch (Larix gmelinii var. japonica)
Source: BMC Plant Biol. 2022 Oct 4;22:470. doi: 10.1186/s12870-022-03862-9 (PMC9531402; doi:10.1186/s12870-022-03862-9)
Supplement: Supplementary file 2 — Additional file 2. Gene ontology (GO) categories in biological process (BP) that encoded proteins with sequence similarity (E-value ≤ 1e-10) in the NCBI database. a) Japanese larch, b) Kuril larch. Gene ontology (GO) categories in cellular component (CC) that encoded proteins with sequence similarity (E-value ≤ 1e-10) in the NCBI database. a) Japanese larch, b) Kuril larch. Gene ontology (GO) categories in molecular function (MF) that encoded proteins with sequence similarity (E-value ≤ 1e-10) in the NCBI database. a) Japanese larch, b) Kuril larch. [file 12870_2022_3862_MOESM2_ESM.pdf]

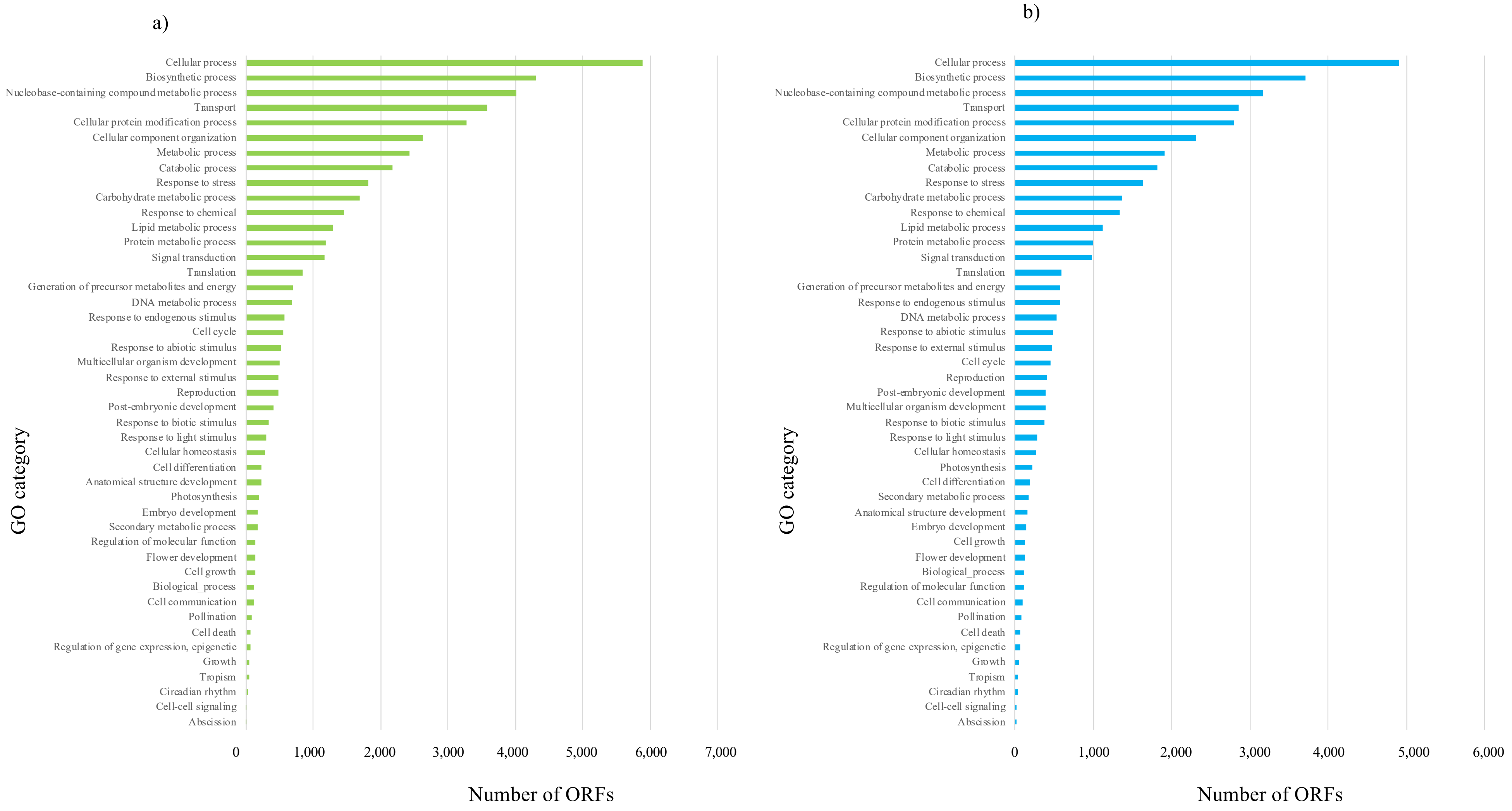

Additional File 2 Gene ontology (GO) categories in biological process (BP) that encoded proteins with sequence similarity (E-value ≤ 1e-10) in the NCBI database. a) Japanese larch, b) Kuril larch.

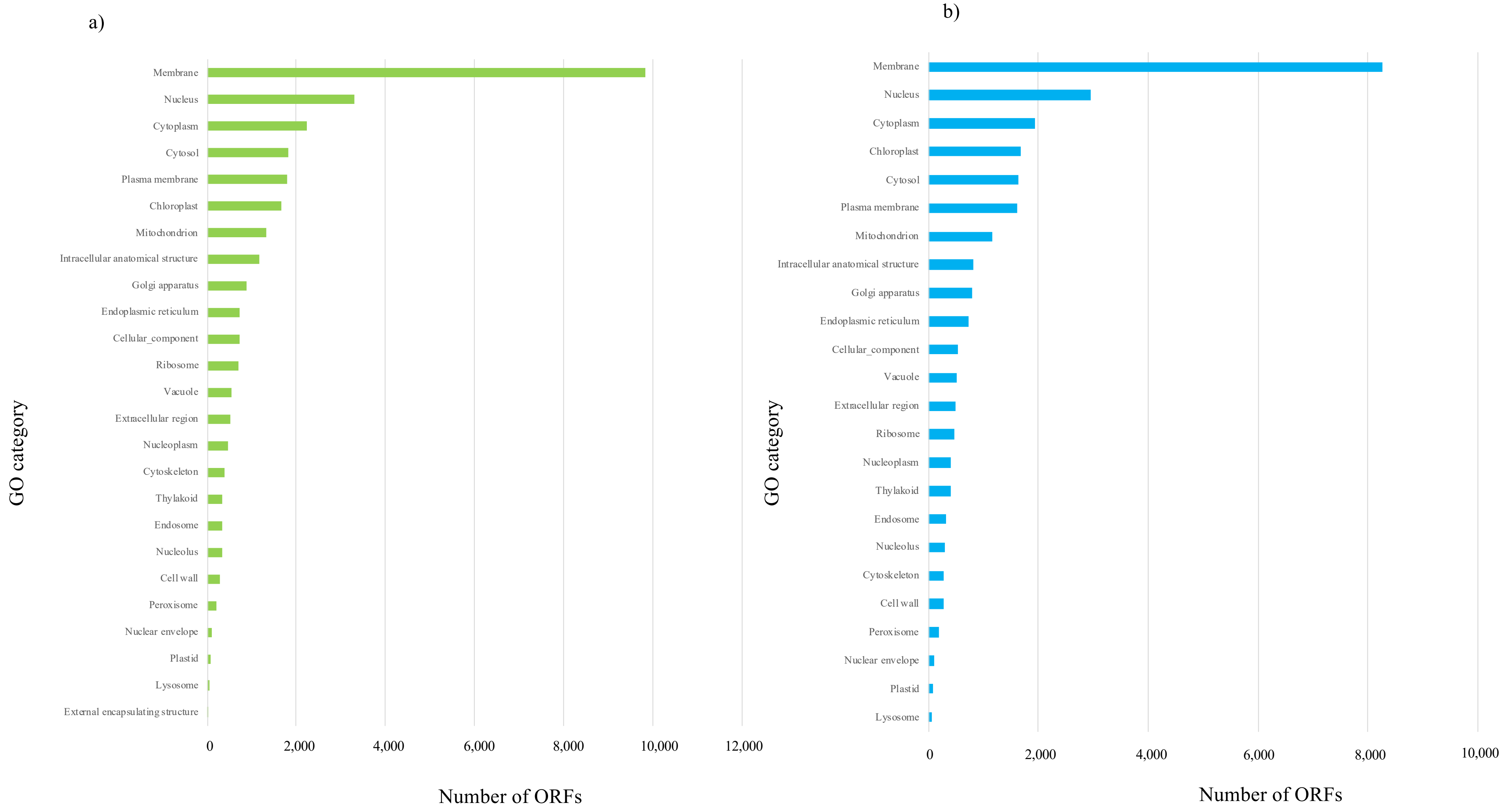

Continued. Gene ontology (GO) categories in cellular component (CC) that encoded proteins with sequence similarity (E-value ≤ 1e-10) in the NCBI database. a) Japanese larch, b) Kuril larch.

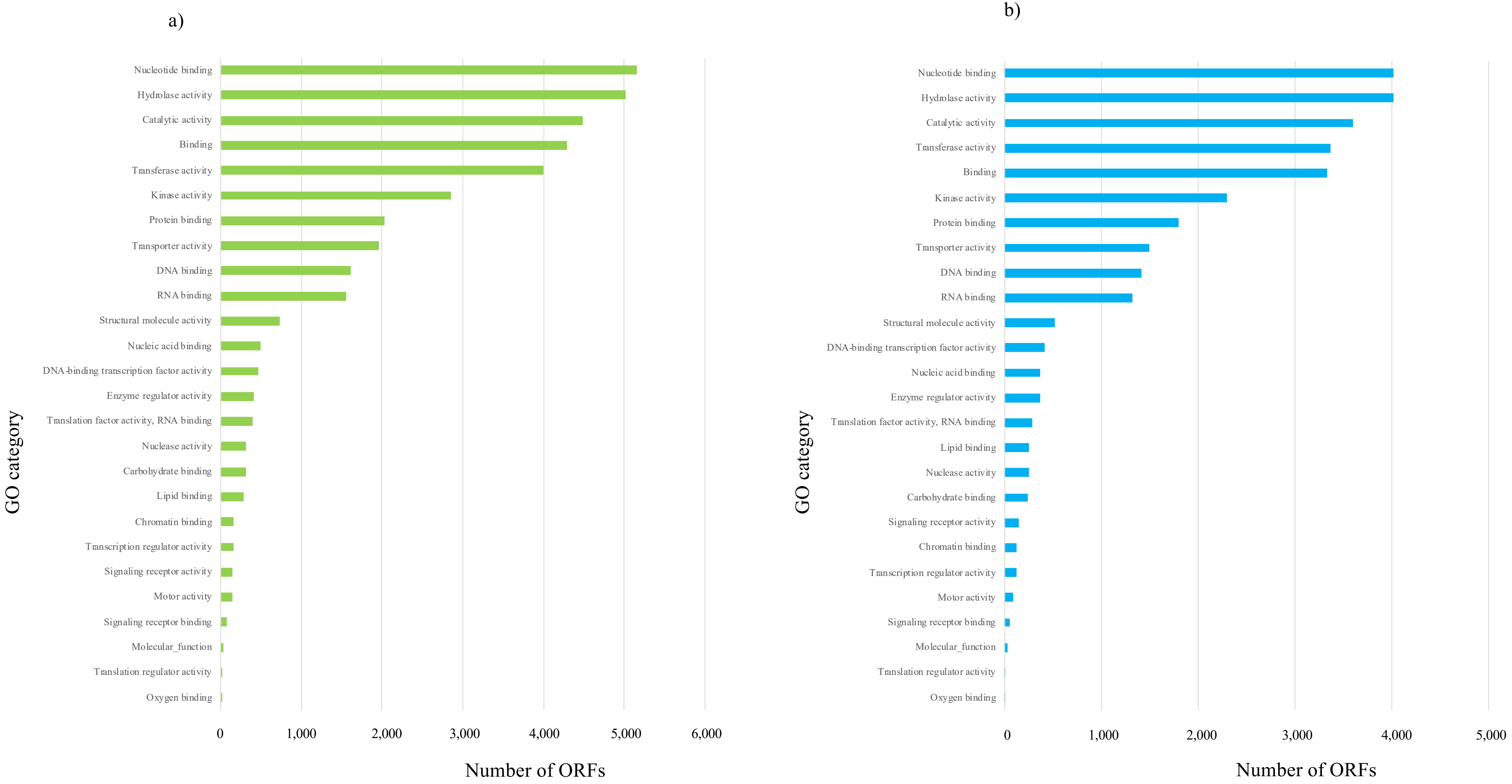

Continued. Gene ontology (GO) categories in molecular function (MF) that encoded proteins with sequence similarity (E-value ≤ 1e-10) in the NCBI database. a) Japanese larch, b) Kuril larch.
