# Supplementary material for: Comprehensive collection of genes and comparative analysis of full-length transcriptome sequences from Japanese larch (Larix kaempferi) and Kuril larch (Larix gmelinii var. japonica)
Source: BMC Plant Biol. 2022 Oct 4;22:470. doi: 10.1186/s12870-022-03862-9 (PMC9531402; doi:10.1186/s12870-022-03862-9)
Supplement: Supplementary file 5 — Additional file 5. Number of open reading frames (ORFs) similar to NCBI sequeces and other species according to BLASTp E-value cutoff values. a) ORFs obtained from Japanese larch, b) ORFs obtained from ORF Kuril larch. [file 12870_2022_3862_MOESM5_ESM.pdf]

a)

b)

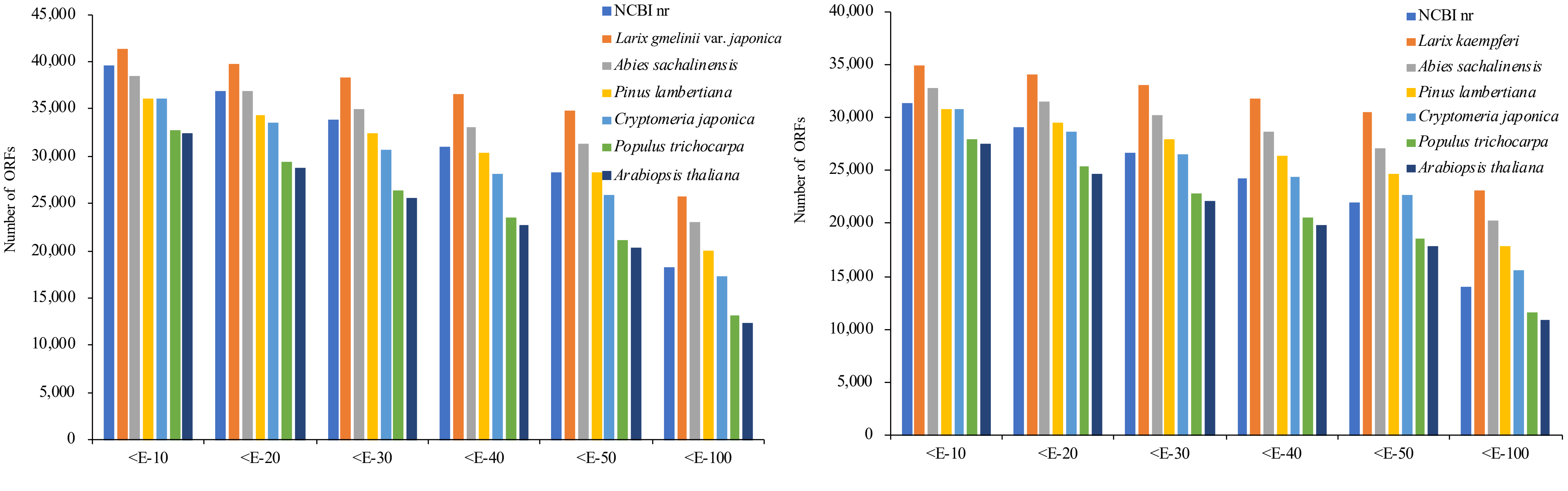

Additional File 5 Number of open reading frames (ORFs) similar to NCBI sequeces and other species according to BLASTp E-value cutoff values. a) ORFs obtained from Japanese larch, b) ORFs obtained from ORF Kuril larch.
