# Supplementary material for: Comprehensive collection of genes and comparative analysis of full-length transcriptome sequences from Japanese larch (Larix kaempferi) and Kuril larch (Larix gmelinii var. japonica)
Source: BMC Plant Biol. 2022 Oct 4;22:470. doi: 10.1186/s12870-022-03862-9 (PMC9531402; doi:10.1186/s12870-022-03862-9)
Supplement: Supplementary file 8 — Additional file 8. Alignment of known FT/FT-like and MFT genes and a set of other angiosperm and gymnosperm sequences. Japanese larch open reading frames are shown in green. Kuril larch open reading frames are shown in blue. [file 12870_2022_3862_MOESM8_ESM.pdf]

	10 20 30 40 50 60 70 80 90 100 110 120 130 140 150 160 170	
Consensus	MMARFVEPLVVGRVIGDVLDMFVPSVDLAVTYGSRQVNNGCEIKPSATVSRPRVDIGGRDLRX--LYTLVMTDPDAPSPSDPTLREYLHWIVTDIPATTDASFGRELXPYEGPRPTIGIHRYIFVLFKQMGRX-TVYPP-QSRNNFSTRDFAEXNGLGLPVAAVYFNAQ-KETAGRRRF	174
Mother of FT and *TFL*-like1: *Pinus sylvestris*(AIJ2001)	-MARSTDPLVVGRVIGDVIDMFVPSMDMAVYYGSKQVTNGCEIKPSATVDRPNVQIAGRHFDES-LYTLVMTDPDAPSPSEPNMREWVHWIVTDIPGATDAAQGREILPYMGPRPPIGIHRYIFVLFKQPGPM-VMMMPPQARNNFSTRAFASQYSLGLPVSAAYFNAQ-KEPGTRKR-	174
MFT-like : *Pinus armandii* (ALH21947)	-MARSTDPLVVGRVIGDVIDMFVPSMDMAVYYGSKQVTNGCEIKPSATVDRPNVQIAGRHFDES-LYTLVMTDPDAPSPSEPNMREWVHWIVTDIPGATDAGQGREILPYMGPRPPIGIHRYIFVLFKQPGPM-VMMMTPQARNNFSTRAFASQNSLGLPVSAAYFNAQ-KEPGTRKR-	174
MFT1-like : *Picea abies* (AEH59565)	-MARSTDPLVVGRVIGDVIDMFVPSNDMAVYYGSKQVRDGCEIKPSATVDRPKVQIAGRHFDDS-LYTLVMTDPDSPSPSEPNMREWVHWVVTDIPGATDAAQGREILPYMGPRPPIGIHRYIFVLFKQSGPM-VMMVPPQARNNFSTRAFASEYSLGLPVSAAYFNAQ-KEPGTRKR-	174
LgMFT-like : LG_T_009821_c00_g01_i01.p1	-MARSTDPLVVGRVIGDVIDMFVPSMDMAVYYGSKQVTNGCEIKPSATVDRPNVQIAGRHFDDS-LYTLVMTDPDAPSPSEPNMREWVHWIVTDIPAATNAAQGREILPYMGPRPPIGIHRYIFVLFKQSGPM-VMTMPPQARNNFSTRAFASQHGLGLPVSATYFNAQ-KEPGTRKR-	174
LkMFT-like : LK_I_c16100_79332	-MARSTDPLVVGRVIGDVIDMFVPSMDMAVYYGSKQVTNGCEIKPSATVDRPNVQIAGRHFDDS-LYTLVMTDPDAPSPSEPNMREWVHWIVTDIPAATNAAQGREILPYMGPRPPIGIHRYIFVLFKQSGPM-VMTMPPQARNNFSTRAFASQHGLGLPVSATYFNAQ-KEPGTRKR-	174
MFT-like: *Ginko biloba* (ANS56341)	-MARSVDPLVVGRVIGDVIDMFTPSVDMAVFYGAKQVTNGCEIKPSATVQRPNVQVAGRHYDDT-LYTLVMTDPDAPSPSEPNMREWIHWMVTDIPGATDATQGKEILPYMGPRPPIGIHRYIFVLFKQRGPM-VMAMPPQQRNNFSIRTFAAQYGLGLPVAAAYFNAQ-KEPGTRKR-	174
MFT2-like: *Picea abies* (AEH59566)	-MARSVDPLVVGNVVGDVIDIFVRAADMTVHYGPKQVTNGCEIKPSATVHRPNLQIAGRHFDDNKLFTLVMTDPDAPSPSEPNMREWLHWIVTDIPGAADASQGREIVPYMGPRPPIGIHRYVFVAFRQQDPM-VMMMAPQVRHNFSTRAFAAQYGLGLPVAAVYFNAQ-KEPANKKR-	175
MFT1: *Arabidopsis thaliana* (NP_173250)	-MAASVDPLVVGRVIGDVLDMFIPTANMSVYFGPKHITNGCEIKPSTAVNPPKVNISGHSDE---LYTLVMTDPDAPSPSEPNMREWVHWIVVDIPGGTNPSRGKEILPYMEPRPPVGIHRYILVLFRQNSPVGLMVQQPPSRANFSTRMFAGHFDLGLPVATVYFNAQ-KEPASRRR-	173
MFT and *TFL1*: *Populus tichocarpa* (XP_002321507)	-MAASVDPLVVGRVIGDVVDMFVPAVKMSVYYGSKHVSNGCDIKPSLSVDPPKVTISGHSDE---LYTLVMTDPDAPSPSEPRMREWVHWIVADIPGGTNPTRGKEILSYVGPRPPVGIHRYILVLFQQKMPLGSMVEPPQNRSHFNTRLYAAHLDLGLPVATVYFNAQ-KEPANKRR-	173
MFT1: *Selaginella_moellendorffii* (FE510546)	-MGRSMDPLVLGRVIGDVLDMFVPAVDMSVCYGSKQVNNGCELKPSATQARPIVQVGSPHEEGA-LYTLVMVDPDAPSPSEPSMREWVHWIVADIPGGADASQGREILQYIGPKPPTGIHRYIFVVFRQMGP--VLMLPPLMRNNFSTRWFAQEYFLGLPVGAVYYNAQ-KEPASRRRT	174
PcFTL1: *Pinus contrta* (GT229995)	-MSRFVEPLVVGRVIGDVLDMFVPGVDLAVTYASRQVNNGCELKPSAVALLPRVDIGGEDLRN--FYTLVMTDPDAPSPSDPTLREYLHWIVTDIPATTSASFGRELVSYESPRPTIGIHRFIFVLFKQIGRQ-TVYPP-GSRINFNTRNFARSQSLGLPVAAVYFNAQ-EETAGRRR-	172
PtFTL1: *Pinus taeda* (jcf7180063169013(Avia et al.2014))	-MSRFVEPLVVGRVIGDVLDMFVPGVDLAVTYASRQVNNGCELKPSAVALLPRVDIGGEDLRN--FYTLVMTDPDAPSPSDPTLREYLHWIVTDIPATTSASFGRELVSYESPRPTIGIHRFIFVLFKQIGRQ-TVYPP-GSRINFNTRNFARSNSLGLPVAAVYFNAQ-KETAGRRR-	172
PaFTL1: *Picea abies* (AEH59567)	-MSRFVEPLVVGRVIGDVLDMFVPSVDLAVTYASRQVNNGCELKPSAITLLPRVDIGGEDLRN--FYTLVMTDPDAPSPSDPTLREYLQWIVTDIPATTSASFGRELVSYESPRPTIGIHRFIFVLFKQMGRQ-TVYPP-GSRLNFNTRNFALSNSLGLPVAAVYFNAQ-KEAAGRRR-	172
PgFTL1: *Picea glauca* (DR546754)	-MSRFVEPLVVGRVIGDVLDMFVPSVDLAVTYASRQVNNGCELKPSALALLPRVDIGGEDLRN--FYTLVMTDPDAPSPSDPTLREYLQWIVTDIPATTSASFGRELVSYESPRPTIGIHRFIFVLFKQMGRQ-TVYPP-GSRLNFNTRNFALSNSLGLPVAAVYFNAQ-KEAAGRRR-	172
LgFTL1: LG_T_045884_c00_g02_i01.p1	-MSRFVEPLVVGRVIGDVLDMFVPSVDLAVTYASRQVNNGCELKPSAVALLPRVDIGGQDLRN--FYTLVMTDPDAPSPSDPTLREYLHWIVTDIPATTSAPFGRELVSYESPRPTIGIHRFIFVLFKQMGRQ-TVYPP-GSRLHFNTRDFAQSNSLGLPVATVYFNAQ-KETAGRRR-	172
LkFTL1: LK_T_019312_c00_g01_i01.p1	-MSRFVEPLVVGRVIGDVLDMFVPSVDLAVTYASRQVNNGCELKPSAVALLPRVDIGGQDLRN--FYTLVMTDPDAPSPSDPTLREYLHWIVTDIPATTSAPFGRELVSYESPRPTIGIHRFIFVLFKQMGRQ-TVYPP-GSRLHFNTRDFAQSNSLGLPVATVYFNAQ-KETAGRRR-	172
FTL1: *Ginko biloba* (EX932314)	-TSRFIEPLAVGRVIGDVLDMFAPSVNLSVIYTSRQVNNGCELKPSAVAISPRVAVGGQDLRT--FYTLVMTDPDAPSPSDPTLREYLHWIVTDIPATTAASFGRELVAYESPRPTIGIHRFVFVLFKQMGRQ-TVYPP-VARQNFNTRSFADLNGLGLPVAAVYFNAQ-KETAGRRR-	172
LgFTL2: LG_I_c00659_00108	-MARFREPLVLGRVIGDVVDMFMPSVNLSATYGSRQVNNGCEIKPSALSSAPRVEVGGDDLRT--CFTLVMTDPDAPSPSDPTLREYLHWIVTDIPATTAASFGRELIRYEAPRPTIGIHRYVFILFKQLARE-TVYPP-QSRINFSTRDFAEMNGLGLPVAAVYYNAQ-KETAPRRRF	173
LkFTL2: LK_I_c41088_26002	-MARFREPLVLGRVIGDVVDMFMPSVNLSATYGSRQVNNGCEIKPSALSSAPRVEVGGDDLRT--CFTLVMTDPDAPSPSDPTLREYLHWIVTDIPATTAASFGRELIRYEAPRPTIGIHRYVFILFKQLARE-TVYPP-QSRINFSTRDFAEMNGLGLPVAAVYYNAQ-KETAPRRRF	173
PaFTL2: *Picea abies* (ABQ85553)	-MARFREPLVLGRVIGDVVDMFMPSVNLTVAYGSRQVNNGCEIKPSAISSAPRVDVGGDDLRT--CFTLIMTDPDAPSPSDPTLREYLHWIVTDIPATTAASFGRELMRYEAPRPTIGIHRYVFTLFKQMARE-TVYPP-QSRVNFSTRDFAEMNGLGLPVAAVYYNAQ-KETAPRRRF	173
PgFTL2: *Picea glauca* (GO363760)	-MARFREPLVLGRVIGDVVDMFMPSVNLTVAYGSRQVNNGCEIKPSAISSAPRVDVGGDDLRT--CFTLIMTDPDAPSPSDPTLREYLHWIVTDIPATTAASFGRELMRYEAPRPTIGIHRYVFTLFKQMARE-TVYPP-QSRVNFSTRDFAEMNGLGLPVAAVYYNAQ-KETAPRRRF	173
PsFTL2: *Pinus sylvestris* (AIJ02007)	-MARFREPLVLGRVIGDVVDMFMPSVNLTVAYGSRQVNNGCEIKPSAISSPPRVDVGGDDLRT--CYTLVMTDPDAPSPSDPTLREYLHWIVTDIPATTAATFGRELMRYEAPRPTIGIHRYVFILFKQMARE-TVYPP-QSRINFSTRDFAEINGLGLPVAAVYYNAQ-KETAPRRRF	173
PtFTL2: *Pinus taeda* (jcf7180063156922(Avia et al.2014))	-MARFREPLVLGRVIGDVVDMFMPSVNLTVAYGSRQVNNGCEIKPSAISSPPRVDVGGDDLRT--CYTLVMTDPDAPSPSDPTLREYLHWIVTDIPATTAATFGRELMRYEAPRPTIGIHRYVFILFKQMARE-TVYPP-QSRINFSTRDFAEINGLGLPVAAVYYNAQ-KETAPRRRF	173
FT: *Arabidopsis thaliana* (Q9SXZ2)	MSINIRDPLIVSRVVGDVLDPFNRSITLKVTYGQREVTNGLDLRPSQVQNKPRVEIGGEDLRN--FYTLVMVDPDVPSPSNPHLREYLHWLVTDIPATTGTTFGNEIVCYENPSPTAGIHRVVFILFRQLGRQ-TVYAP-GWRQNFNTREFAEIYNLGLPVAAVFYNCQRESGCGGRRL	175
TFL1: *Arabidopsis thaliana* (NP_196004)	-GTRVIEPLIMGRVVGDVLDFFTPTTKMNVSYNKKQVSNGHELFPSSVSSKPRVEIHGGDLRS--FFTLVMIDPDVPGPSDPFLKEHLHWIVTNIPGTTDATFGKEVVSYELPRPSIGIHRFVFVLFRQKQRR-VIFPNIPSRDHFNTRKFAVEYDLGLPVAAVFFNAQ-RETAARKR-	173
MFT2: *Selaginella_moellendorffii* (FE451588)	-DVNALDPLILGGIIPDVVDDFVPCCEMAVYYGKDQVTNGCELAPFATSSPPNVQIAG-NFDDGSLFTLVMTDPDAPSPAEPSLGEYLHWLVTDIPGGTDPSKGKGVLPYERPKPPAGTHRYTFCLFKQSRPM--MALAPVIRSNFSTKCFAQEHGLGLAVAALYFKAQGMEP------	169

Additional File 8 Alignment of known *FT/FT*-like and *MFT* genes and a set of other angiosperm and gymnosperm sequences. Japanese larch open reading frames are shown in green. Kuril larch open reading frames are shown in blue.
