# Supplementary material for: Comprehensive collection of genes and comparative analysis of full-length transcriptome sequences from Japanese larch (Larix kaempferi) and Kuril larch (Larix gmelinii var. japonica)
Source: BMC Plant Biol. 2022 Oct 4;22:470. doi: 10.1186/s12870-022-03862-9 (PMC9531402; doi:10.1186/s12870-022-03862-9)
Supplement: Supplementary file 9 — Additional file 9. Alignment of known CONSTANS genes and a set of other angiosperm and gymnosperm sequences. Japanese larch open reading frames are shown in green. Kuril larch open reading frames are shown in blue. [file 12870_2022_3862_MOESM9_ESM.pdf]

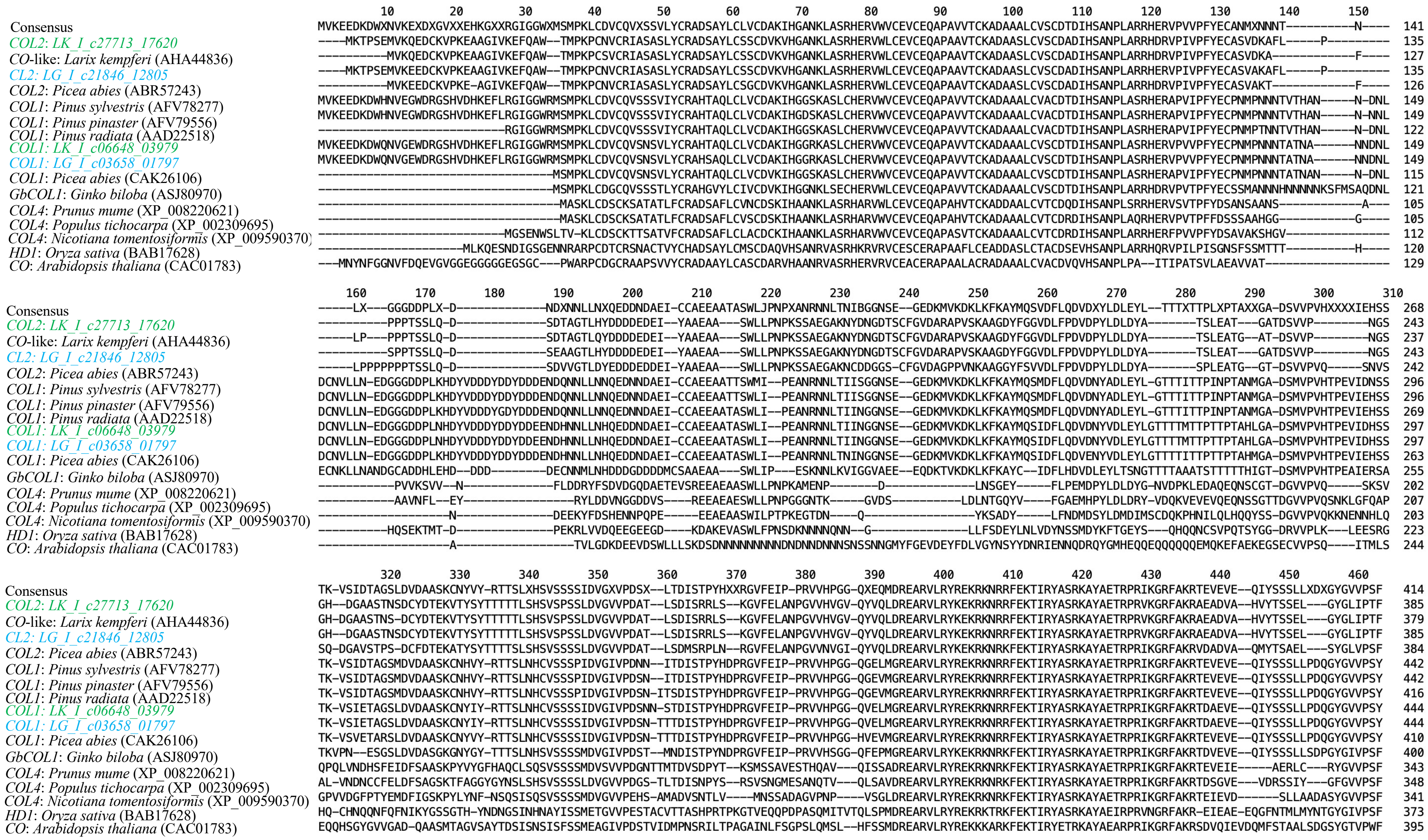

Additional File 9 Alignment of known *CONSTANS* genes and a set of other angiosperm and gymnosperm sequences. Japanese larch open reading frames are shown in green. Kuril larch open reading frames are shown in blue.
