# Supplementary figures and images for: Comprehensive collection of genes and comparative analysis of full-length transcriptome sequences from Japanese larch (Larix kaempferi) and Kuril larch (Larix gmelinii var. japonica)
Source: BMC Plant Biol. 2022 Oct 4;22:470. doi: 10.1186/s12870-022-03862-9 (PMC9531402; doi:10.1186/s12870-022-03862-9)

## ***Isoform sequence (PacBio RSII)***

## ***Short-read sequence (Illumina HiSeq)***

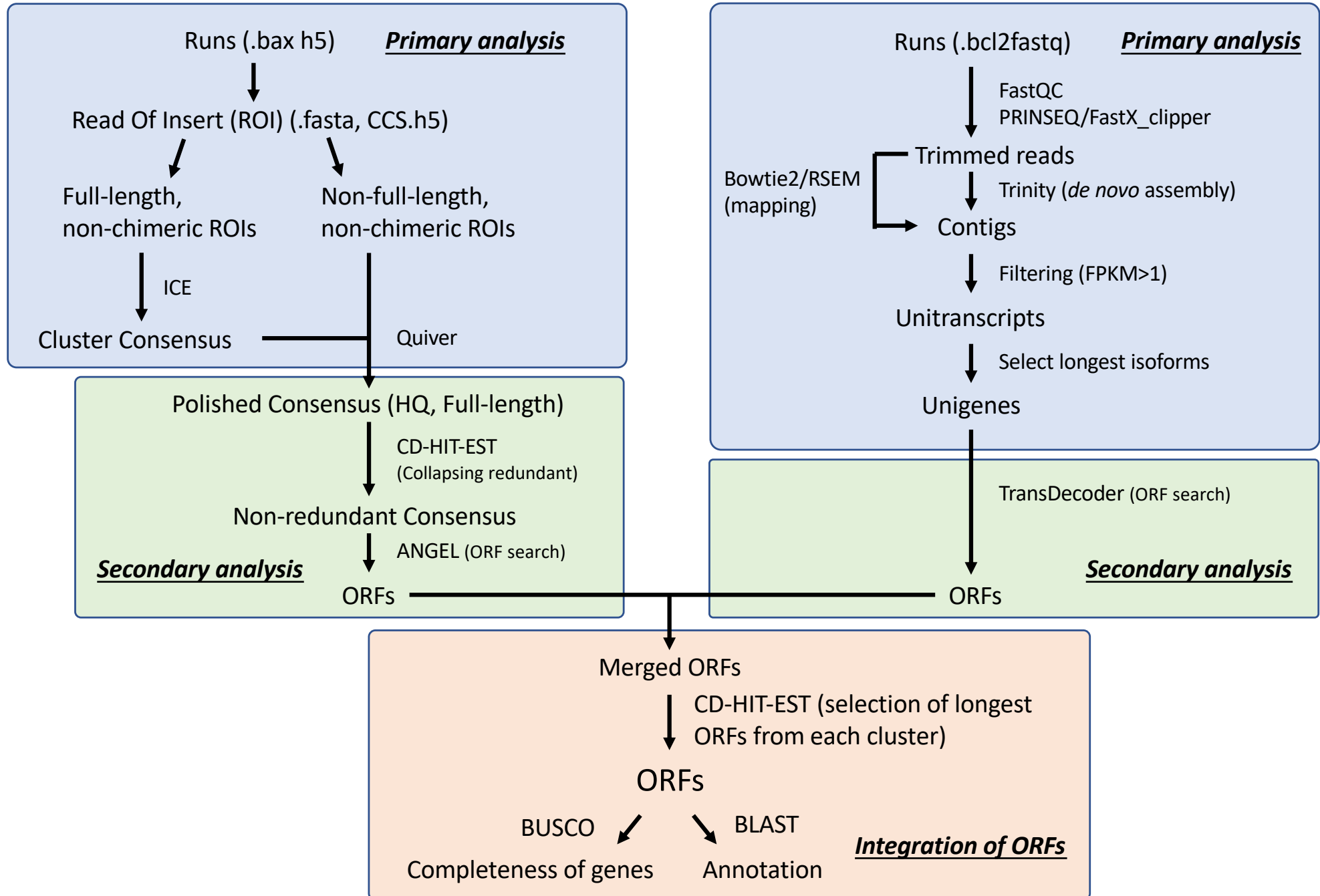

Supplement: Supplementary file 11 — Additional file 11. Summary of assembly and characterization. [file 12870_2022_3862_MOESM11_ESM.pdf]
